# Recent Advancements in Sodium Alginate-Based Hydrogels Combined with Magnetic Nanoparticles for Biological Applications: A Review

**DOI:** 10.3390/gels12060508

**Published:** 2026-06-08

**Authors:** Kun Fang, Pei Li, Xiangrui Huang, Hanbing Wang, Yihan Li

**Affiliations:** 1 Dabie Mountain Laboratory, College of Tea and Food Science, Xinyang Normal University, Xinyang 464000, Chinalyh060629@126.com (Y.L.); 2 Henan Key Laboratory of Tea Plant Biology, Xinyang Key Laboratory of Food Innovation for Tea and *Camellia oleifera*, Xinyang 464000, China; 3 Huaihe Campus Administrative Committee, Xinyang Normal University, Xinyang 464000, China; lipei@xynu.edu.cn

**Keywords:** sodium alginate, magnetic nanoparticles, hydrogels, biological applications

## Abstract

The emergence of organic–inorganic hybrid composites integrating magnetic nanoparticles (MNPs) with polymers has been an important advancement in modern biological research. Among these systems, magnetic sodium alginate (SA)-based hydrogels (MSABHs), produced by embedding MNPs within an SA framework, exhibit remarkable potential for biomedical applications owing to their high biocompatibility, rapid magnetic response, controllable spatiotemporal behavior, and remote, non-invasive operation. Under the influence of an alternating magnetic field (AMF), MSABHs can exhibit various responses, including deformation, motion, and thermal generation, which are highly valuable for diagnostic and therapeutic medical applications. This review first outlines the key studies on SA and MNPs, along with the various synthesis routes used to fabricate MSABHs. Subsequently, the discussion primarily focuses on their versatile biomedical applications, including tissue engineering, targeted drug delivery, thermotherapy, imaging, and micro-robotics, followed by an evaluation of current challenges and prospects for future improvement. Through this comprehensive examination and synthesis, the review aims to further reveal the full potential of MSABHs and broaden their applications in the biological domain.

## 1. Introduction

With the growing worldwide focus on promoting health, biomedicine has become an essential domain of scientific and technological progress [1]. As the demand for biomedical strategies to address diverse diseases continues to rise, the development of biocompatible materials with advanced, innovative biological functions has become increasingly urgent [2]. In this context, magnetic nanoparticles (MNPs) have rapidly gained recognition as among the most significant functional nanomaterials used in biomedical research and applications [3]. Owing to their nanoscale structure, superior cellular permeability, and magnetic responsiveness, MNPs have been extensively employed in thermotherapy [4], drug delivery [5], medical imaging [6], tissue engineering [7], and therapeutic interventions [8]. Typically, MNPs consist of metallic elements such as cobalt (Co), iron (Fe), nickel (Ni), and their corresponding oxides [9]. Among these, Fe_3_O_4_ nanoparticles (NPs) are particularly prominent in biomedical applications due to their superparamagnetic behavior, stable physicochemical properties, compatibility with biological systems, and minimal toxicity [10]. Nevertheless, isolated MNPs are susceptible to agglomeration as a result of magnetic dipole–dipole attractions, oxidation tendency, and the activity of surface groups located on their monoclinic crystalline planes [11]. To improve stability and enable targeted surface modifications, numerous surface functionalization methods have been developed.

Polymers containing active functional groups, such as hydroxyl (-OH), can readily interact with MNPs, serving as key functionalization agents that significantly influence their pharmacokinetics and biodistribution [12]. These polymers may originate from synthetic sources, polyvinyl alcohol [13], polystyrene [14], and polyethylene glycol [15]; or natural polymers, including lignin [16], cellulose [17], SA [18], chitosan [19], starch [20], and protein [21]. SA, a linear natural polysaccharide extracted from brown seaweed, is composed of β-D-galactose (G) and α-L-mannose (M) residues connected through 1 → 4 glycosidic linkages, forming distinctive MM, GM, and GG block sequences [22]. SA is water-soluble, non-toxic, biocompatible, biodegradable, and non-immunogenic. It has been applied in various biomedical fields, with the most promising applications including drug delivery, gene delivery, and wound dressings [23]. Its molecular chain is enriched with hydroxyl (-OH) and carboxyl (-COOH) groups, which enable chelation with divalent and trivalent metal ions such as Ca^2+^, Fe^3+^, Cu^2+^, Ba^2+^, and Al^3+^, generating a characteristic “egg-box” structured hydrogel network [24]. Moreover, SA can undergo structural modification via chemical, biological, or physical approaches to acquire desirable physicochemical properties, making it a good carrier or template for MNPs [25]. The integration of MNPs with SA, therefore, combines the beneficial features of both polymeric and magnetic materials. Specifically, while MNP incorporation improves the mechanical stability of SA and introduces magnetic targeting, thermo-responsiveness, and facile separation properties [26], the SA matrix simultaneously provides the MNPs with enhanced surface activity and superior biocompatibility [27].

An examination of research publications from the past ten years using the keyword combination “magnetic SA biomedical” in Google Scholar indicates a remarkable growth in the number of related studies (Figure S1). This upward trend highlights the growing research interest and promising potential of magnetic SA-based hydrogels (MSABHs), which are essential for advancing high-quality biomedical materials. Although considerable achievements have been made, investigations into MSABHs are still at a relatively early stage, and their full biomedical potential remains largely untapped. To accelerate knowledge integration and support continued innovation in this rapidly evolving domain, a systematic review of the most recent advances in MSABHs is essential. To date, most reviews have focused on the individual biomedical uses of SA or MNPs, while comprehensive analyses specifically devoted to MSABHs and their biological applications are still scarce. Therefore, undertaking an in-depth review of MSABHs is vital to enhance their future development and utilization in biomedical science. This review provides an overview of MSABHS synthesis methodologies and a detailed discussion of their diverse biomedical applications. Furthermore, it identifies the main obstacles limiting their current practical use and proposes future research directions. Considering the distinctive characteristics and notable advantages of MSABHs in biological contexts, this review aims to strengthen understanding of emerging research trends and technological innovations aimed at producing high-performance, sustainable, and cost-efficient MSABHs, thereby fostering their continued progress in biomedical fields.

## 2. SA’s Fundamental Background

### 2.1. SA’s Sources and Extraction

SA is a natural polysaccharide derived from the cell walls of brown algae, where it forms a gel-like matrix with divalent metal salts [28]. Its hydroxyl-rich chain structure readily forms strong hydrogen bonds, which limit its water solubility and practical applications. Therefore, alginic acid is typically converted into salt forms, with alginate being the most stable and widely used derivative [29]. In commercial production, SA is extracted from various brown algae, including the genera Laminaria, Sargassum, Durvillaea, Macrocystis, and Ecklonia, and the families Rhodophyceae and Phaeophyceae [30]. Due to its higher yield and quality, Sargassum is typically the preferred source for industrial extraction [31]. Algae that produce alginic acid also include various macroalgae, as well as less common but specialized species, such as Japanese seaweed [32]. Regional variations in seaweed composition and growth conditions can affect the yield and purity of alginic acid [33]. Furthermore, SA produced by bacterial fermentation (e.g., nitrogen-fixing bacteria and Pseudomonas) exhibits superior purity and uniformity compared to seaweed-derived SA [34]. Among the studied strains (*Pseudomonas acetica*, *Pseudomonas aeruginosa*, *Pseudomonas fluorescens*, *Pseudomonas mendocina*, *Pseudomonas cinnamoma*, *Pseudomonas* spp., and *Agrobacterium venneri*), *Pseudomonas cinnamoma* is the most efficient strain for industrial SA production [35].

The extraction of alginate follows a standard procedure: washing, formaldehyde treatment (cross-linking and decolorization), hydrochloric acid treatment (removal of phenolic compounds and conversion of insoluble alginate to a soluble form), alkaline extraction (adjusting properties by controlling solvent concentration, temperature, time, and pH), neutralization, precipitation, filtration, drying, and grinding [36,37]. Microbial fermentation has emerged as an alternative method, yielding bacterial-derived alginate with more stable physicochemical properties [38]. The degree of polymerization of alginate ranges from approximately 50 to 3000, corresponding to molecular weights of 10–600 kDa [39]. These characteristics depend on the biological source, algal species, and extraction parameters [40].

### 2.2. Structure of SA

SA is a copolymer formed by β-D-mannuronic acid (M) and α-L-guluronic acid (G) linked via 1 → 4 glycosidic bonds, forming MM, GG, and GM units [41]. The proportions and arrangements of these units determine the properties of SA. Hecht et al. [42] reported its chain conformation (Figure S2a). SA has a molecular weight of 20,000–600,000 and a degree of polymerization of 100–3000 [43]. The mean-squared end-to-end distance of GG is 2.2 times that of MM [44]. Rigid G residues enhance cross-linking and stability, while flexible M residues provide elasticity [45,46]. Consequently, the monomer distribution determines viscosity, ion selectivity, and gelation properties [47]. The M/G ratio varies by source (feathers have a higher M content, while fixators are richer in G) [48,49]. Hydroxyl groups at the C_2_/C_3_ positions and carboxyl groups at the C6 position enable selective modification [50].

^1^H and ^13^C nuclear magnetic resonance (NMR) provide detailed structural information. Penman et al. [51] described a method for determining the M/G ratio via ^1^H NMR by separating the homogeneous and alternating components through partial acid hydrolysis. Grasdalen et al. [52] correlated the molar M/G ratio with ^1^H NMR signal intensity. Representative isomer regions are shown in Figure S2b. The mole fraction of M acid follows FG + FM = 1 [53], with FGG + FGM = FG and FMM + FMG = FM [54]. For alginate chains of sufficient length, typically those with an average degree of polymerization greater than 20, the effects of terminal reducing residues can be neglected. Under this assumption, FMG is considered equal to FGM, which enables calculation of both the numerical M/G ratio and the corresponding doublet peak frequencies [55].

Alginate forms physical gels with cations. Their mechanical properties depend on the type of cation and the polymer sequence [56]. As the G content increases, stiffness increases; homopolymers are stiffer than heteropolymers [57]. Ca^2+^ promotes cross-linking, but the chain composition determines stiffness [58]. A higher M content increases flexibility. The G content influences interchain interactions (lateral, zipper-like, and entanglement) [59]. M residues act as elasticity modulators. As shown in Figure S2c, copolymers are more flexible than homopolymers regardless of G content; at higher concentrations, they reduce interactions and promote cross-linking. Chain length, sequence, and concentration collectively determine SA’s behavior [60].

### 2.3. Properties of SA

Recent wide studies on applications of SA have provided a deeper understanding of its structure–property relationships. The material characteristics of SA are primarily governed by the arrangement and proportion of urate residues, the polymer’s molecular weight, and the concentration of the cationic solution used for cross-linking [61].

#### 2.3.1. Solubility

SA is highly water-soluble but nearly insoluble in organic solvents. Solubility depends on several factors: (a) polymer sequence—SA with more G blocks dissolves more readily than M-rich ones [62]; under acidic conditions, alginates with balanced M/G remain soluble, while those with segregated blocks do not [63,64]; (b) solution pH—deprotonation of carboxyl groups above a threshold enables dissolution [65]; (c) ionic strength—in the absence of gelling ions, higher ionic strength increases solubility by electrostatic shielding, reducing chain entanglement and viscosity [66]; (d) divalent cations (Ba^2+^, Ca^2+^, Sr^2+^)—they promote gelation and hinder solubility, so SA needs a solvent free of cross-linking ions [67]. Moreover, fully protonated carboxyl groups make SA insoluble even in water [68]. The negatively charged groups in SA solutions also help condense hydrophobic suspensions and contribute to anti-charge behavior.

#### 2.3.2. Viscosity

SA undergoes substantial swelling when dispersed in aqueous media, with its absorption capacity reaching up to ten times its original volume. This pronounced swelling significantly increases solution viscosity, which is why experimental preparations typically limit SA concentrations to 10% *w*/*w* or lower [69]. Alginate samples enriched in G residues exhibit better solubility than those with higher M content [70]. The viscosity of SA solutions also depends on both the chain length and the total number of monomeric units. At the same concentration, SA with longer polymer chains generally exhibits higher viscosity, indicating that molecular weight directly influences the polymer’s rheological properties [71]. In contrast, bacterial-derived SA does not demonstrate a similar correlation between chain length and solution viscosity [72].

#### 2.3.3. Ion-Induced Gelation

The gelation process of SA occurs under the action of cations such as Cu^2+^, Fe^3+^, Ca^2+^, Ba^2+^, Al^3+^, and H^+^; it is driven by the electrostatic attraction between the cations and the negatively charged carboxyl groups (CGs) of sialic acid [73]. The gel formed varies depending on the cation used. Below the pKa of the carboxylate residues, H^+^ can form a soft, water-soluble gel, although slight pH variations during the gelation process can affect the uniformity of the gel [74]. Divalent cations are widely used for strong gelation. Ba^2+^ can cross-link alginate microgels and contribute to the formation of BaSO_4_ nanoparticles within the matrix [75]. Ba^2+^ has a higher affinity for alginate than Ca^2+^, allowing for the formation of stronger and more stable gels [76]. BaSO_4_/alginate microgels also exhibit good radiographic contrast, making them suitable for arterial embolization therapy. The order of affinity of alginate for divalent cations has been reported as follows: Pb^2+^ > Cu^2+^ > Cd^2+^ > Ba^2+^ > Sr^2+^ > Ca^2+^ > Co^2+^ > Ni^2+^ > Zn^2+^ > Mn^2+^ [77]. Among these, Ca^2+^ is particularly notable for gel formation. It replaces H^+^ and Na^+^ ions in alginate to form a Ca–SA network, in which the ions integrate into an “egg-box” structure composed of G units [78] (Figure S3a). This egg-box model was initially proposed by Grant et al. [79]. Depending on how Ca^2+^ is added, gelation can be classified as external gelation or internal gelation (Figure S3b) [80].

For monovalent ions, gelation depends on the degree of CG protonation. Divalent ions exhibit different affinities for G, M, and MG units, whereas trivalent ions show no selectivity. Consequently, gels prepared from ions of different valences differ in terms of formation mechanism, mechanical strength, viscoelasticity, and biocompatibility.

### 2.4. Modifications of SA

The properties of SA are not always sufficient, necessitating modification of its physicochemical characteristics. There are three approaches: physical [81], chemical [82], and biological [83]. Physical modification: this includes cross-linking and blending. Cross-linking involves hydrogen bonds, microcrystals, or entanglements [84]. Cross-linking via Ca^2+^ results in an “egg-box” structure [85]. Blending sialic acid with polysaccharides [86,87], lipids [88], or other compounds via hydrogen bonds preserves the benefits of sialic acid while adding new ones. Blends with chitosan [89,90] or gelatin [91] often improve mechanical strength, antioxidant activity, and antimicrobial effects. Chemical modification: utilizes free hydroxyl and carboxyl groups to modify the network structure, adjusting hydrophilicity and covalent integrity [92]. Common methods include oxidation, esterification, amidation, graft copolymerization, and cross-linking (Figure S4) [93]. Modified SA exhibits improved mechanical strength, increased surface area, and greater activity. Biological modification: uses SA lyase to break down SA into oligosaccharides via β-elimination [94]. The reaction removes the negative charge from the carboxyl group, extracts the C5 proton (alkali), and then cleaves the 4-O-glycosidic bond to form a C4=C5 double bond. M residues yield a cis configuration, while G residues yield a trans configuration. Lyases are of exo- or endo-type [95]. After modification, many of SA’s properties have changed, making it more suitable for biological applications.

### 2.5. Limitations of SA

Several inherent drawbacks limit the application of SA. Its porous and degradable network exhibits insufficient mechanical strength and can lead to premature release of substances encapsulated within the matrix [96]. Additionally, SA gels tend to shrink under acidic conditions, reducing their effectiveness in retaining water-soluble compounds (e.g., pharmaceuticals) and causing increased leakage from Ca^2+^-crosslinked SA networks [97]. Gel formation also exhibits hysteresis due to electrostatic interactions between the COO^−^ groups of SA and Ca^2+^ ions [98]. Other challenges include rapid degradation, poor solubility in organic solvents, a lack of active cell-adhesion sites, and complex extraction procedures. Moreover, SA is further susceptible to degradation in strongly acidic or alkaline environments, demonstrates limited selectivity for backbone modifications, is sensitive to high temperatures, and may yield products with inconsistent molecular weights [99]. To overcome these limitations, MNPs are typically incorporated to not only enhance the mechanical and structural properties of SA but also to impart magnetic properties to the composite material. Specifically, the introduction of magnetic MNPs not only improves the mechanical stability of SA but also enhances its ease of separation. At the same time, the SA matrix provides the MNPs with enhanced surface activity and excellent biocompatibility.

## 3. Types of MNPs in MSABHs

The magnetic properties of MSABHs are influenced by multiple factors, including the content, distribution, type, and size of MNPs, as well as their interactions with the SA matrix, which in turn affect their biological applications. MNPs are generally composed of metals such as cobalt, nickel, and iron, as well as their oxides [100]. Common forms of MNPs include Fe_3_O_4_, Fe_2_O_3_, and AB_2_O_4_. Among these, Fe_2_O_3_ exists in four prismatic polymorphs, each exhibiting unique structural and magnetic features: (i) α-Fe_2_O_3_, (ii) β-Fe_2_O_3_, (iii) γ-Fe_2_O_3_, and (iv) ε-Fe_2_O_3_. It has also been observed that subjecting α-Fe_2_O_3_ to extremely high pressures can induce the formation of a novel perovskite structure. Of these, γ-Fe_2_O_3_ and α-Fe_2_O_3_ occur naturally, whereas ε-Fe_2_O_3_ and β-Fe_2_O_3_ are typically synthesized in laboratories, along with NPs of diverse structural morphologies. The primary crystal structures of Fe_2_O_3_, (a) α-Fe_2_O_3_, (b) β-Fe_2_O_3_, (c) γ-Fe_2_O_3_, and (d) ε-Fe_2_O_3_, are schematically illustrated in Figure S5 [101].

As illustrated in Figure S6a, Fe_3_O_4_ exhibits a face-centered cubic spinel structure built upon 32 O^2−^ ions, which are densely packed along the (111) direction. Unlike many other iron oxides, Fe_3_O_4_ contains both divalent and trivalent iron ions. It forms a cubic inverse spinel structure in which oxide ions are arranged in a cubic close-packed lattice: Fe^2+^ ions occupy half of the octahedral sites. In contrast, Fe^3+^ ions are distributed across the remaining octahedral and tetrahedral positions [102]. Additionally, spinel metal oxides (AB_2_O_4_) are highly promising for biomedical applications due to their small particle size, high surface-to-volume ratio, and magnetic properties. In AB_2_O_4_, A and B denote metal cations positioned at octahedral (B site) and tetrahedral (A site) locations, as shown in Figure S6b [103]. Typically, the A site hosts divalent metals such as Co, Zn, Mg, or Mn, whereas the B site is occupied by trivalent metals like Al or Fe [104].

In general, various MNPs, including spinel ferrites such as MgFe_2_O_4_ and CoFe_2_O_4_, as well as iron oxides like Fe_2_O_3_ and Fe_3_O_4_, can be added into MSABHs to serve as magnetically responsive components. And, among these, Fe_3_O_4_ is the most commonly used iron oxide in biomedical applications due to its high magnetization, ease of synthesis, good biocompatibility, and environmental safety [105]. Fe_3_O_4_ demonstrates improved magnetization, especially when decreased to single-domain NPs. These MNPs can be readily manipulated or recovered using an AMF and exhibit no residual magnetic interactions upon field termination.

## 4. Synthesis Strategies of MSABHs

**Figure 1:**
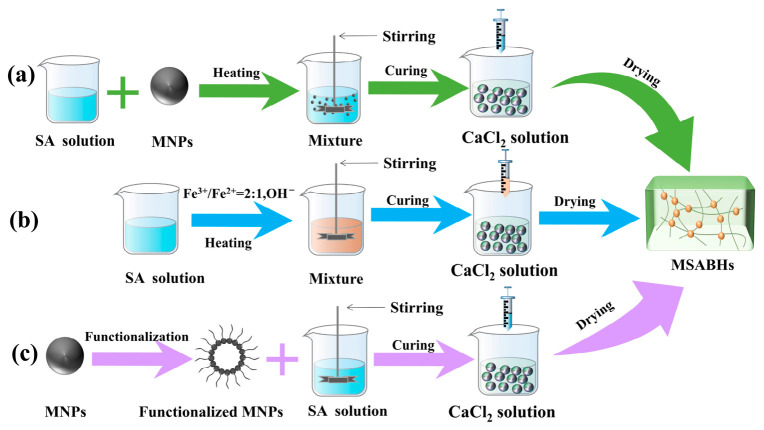
Different preparation strategies for MSABHs. (**a**) The SA slurry is mixed with the MNPs suspension and then gelatinized, allowing the nanoparticles to be embedded within the matrix (blending approach). (**b**) A SA network is first produced, after which iron ions are adsorbed into the polymer framework and subsequently converted to nanoparticles through alkaline in situ coprecipitation (in situ coprecipitation approach). (**c**) MSABHs are generated using surface-functionalized MNPs as crosslinking agents (grafting approach).

Interactions between MNPs and the SA matrix are generally weak. Nevertheless, MNPs’ functionalization can substantially strengthen these interactions, thereby enhancing the overall features of MSABHs. The fabrication of MSABHs typically employs three main strategies: in situ coprecipitation, blending, and grafting. In the blending method, MNPs are physically dispersed within the SA matrix, whereas in grafting, chemical bonds form between MNPs and SA chains. Although the in situ coprecipitation approach is more economical, it may lead to partial degradation of the SA matrix during synthesis. These strategies can be applied individually or in combination to generate MSABHs with tailored physicochemical characteristics. Therefore, careful selection of materials and synthetic approaches is crucial for designing MSABHs with specific functional properties. Figure 1 presents a simplified illustration of four MSABHS synthesis routes: blending, in situ coprecipitation, and grafting.

### 4.1. Blending Method

The blending technique is widely employed for the fabrication of MSABHs. This approach involves combining MNPs with the SA matrix, adding water, and then applying heat and stirring. Intermolecular interactions, such as hydrogen bonding, electrostatic interactions, and van der Waals forces, contribute to the formation of a stable and ordered composite structure. Before blending, MNPs are typically synthesized using methods such as coprecipitation, hydrothermal synthesis, sol–gel processing, or pyrolysis. For instance, Zhang et al. [106] encapsulated calcium peroxide (CP) within polylactic acid microspheres and blended these with bone marrow stromal cells (BMSCs) and MNPs in a SA solution. The mixture was then delivered via a peristaltic pump into a calcium chloride solution, yielding a sustainable alginate microcarrier that combines oxygen-generating capacity with magnetic responsiveness. The release of calcium ions created weak interactions with SA, improving structural stability and providing support for BMSCs, thereby establishing a microenvironment more closely resembling in vivo conditions. Moreover, blends prepared via this method can be used in 3D or 4D printing of MSABHs. In a previous study, Simińska-Stanny et al. [107] introduced a four-dimensional printing approach to fabricate patterned magnetic hydrogel constructs from natural polymers. As shown in Figure S7b(1), the magnetic ink contained SA, methylcellulose (MC), and Fe_3_O_4_ NPs stabilized by polyacrylic acid (PAA). By adjusting the relative concentrations of these three components (Figure S7b(2)), three-dimensional structures, including tubular (wheel-like), cubic, and cantilever beam geometries, were successfully printed (Figure S7b(3)). Programmable patterning enabled the creation of macroanisotropic magnetic hydrogels that demonstrated shape- and pattern-dependent magnetic responses, including rolling, bending, and jumping, when exposed to an AMF (Figure S8b(4)).

The blending approach typically involves two main stages: the synthesis of MNPs, followed by the SA matrix solution’s preparation. Due to the wide range of techniques available for both MNP fabrication and SA solution preparation, it is probable to precisely control the size, content, and functional properties of MNPs, as well as the concentration and composition of the SA matrix. This versatility allows the final MSABHs to be tailored to specific desired characteristics. Furthermore, the method is relatively simple and adaptable for diverse applications. However, a common limitation of this approach is the tendency of MNPs to aggregate, leading to uneven dispersion within the SA matrix.

### 4.2. In Situ Co-Precipitation Method

Co-precipitation is a rapid and convenient method for producing MNPs from Fe^2+^/Fe^3+^ aqueous solutions by adding an alkaline precipitating agent, such as NH_3_·H_2_O or NaOH, at ambient or elevated temperatures, typically under an inert atmosphere [108]. This in situ co-precipitation technique can also be used to fabricate MSABHs using SA solutions, in which the SA matrix serves as a chemical reactor for nanoparticle formation. For example, Benjamin et al. [109] dissolved SA powder in water and stirred the mixture to obtain a transparent solution. A solution containing Fe^2+^ and Fe^3+^ salts in solvent was then added dropwise to the polymer solution at a 1:2 molar ratio, forming a pregel mixture. After stirring, co-precipitation of the iron salts was initiated with NaOH to maintain a pH of 12, yielding a black magnetic nanoparticle suspension of iron oxide. The reaction proceeded at room temperature, after which the alginate-coated iron oxide NPs (SA-Fe_3_O_4_) were washed repeatedly with water and ethanol until the pH was neutral. The SA-Fe_3_O_4_ particles were further conjugated with L-tryptophan via the EDC-NHS pathway, producing a material with good superparamagnetic properties and potential scavenging activity, as illustrated in Figure S8a. In another study, Eivazzadeh-Keihan et al. [110] added FeCl_3_·6H_2_O and FeCl_2_·4H_2_O solutions to a gel matrix under mechanical stirring in a nitrogen atmosphere. Aqueous ammonia was then added in portions to trigger co-precipitation. Upon completion, the mixture was cooled, and the resulting black magnetic SA-tannic acid (TA)/silk fibroin (SF)/Fe_3_O_4_ nanocomposites were collected using an external magnet, as shown in Figure S8b.

The in situ co-precipitation technique provides a simple, economical route to achieve well-distributed MNPs throughout the SA network. Nevertheless, the alkaline environment required for this process may be overly aggressive for certain SA matrices, particularly those that are unstable in alkaline media. For instance, ester bonds may hydrolyze at pH levels above 10. In addition, the use of MSABHs in aqueous environments can result in leakage and diffusion of MNPs.

### 4.3. Grafting Method

The grafting approach involves separately preparing MNPs and the SA matrix, with functionalized MNPs serving as cross-linkers that provide covalent attachment sites within the SA network. Therefore, functionalization of the NPs is crucial to introduce reactive groups onto their surfaces. For example, Tahmasebi et al. [111] dispersed Fe_3_O_4_ NPs in distilled water using ultrasonic treatment. Subsequently, silver NPs were added to the Fe_3_O_4_ suspension under magnetic stirring. The resulting mixture was then utilized to synthesize the bio-nanocomposite hydrogel. Meanwhile, SA was dissolved in distilled water, and β-cyclodextrin was added with stirring. The modified iron oxide NPs were incorporated into this solution at room temperature and stirred. To form magnetic beads, the SA/β-CD/Fe_3_O_4_@Ag solution was injected dropwise into calcium chloride solution using a syringe. Additional stirring ensured robust internal cross-linking within the hydrogel microspheres. The final magnetic microspheres were repeatedly washed with distilled water and dried, as shown in Figure S9a. In another study, a three-step grafting procedure was proposed for the synthesis of MSABHs, as shown in Figure S9b [112]. The procedure includes: (1) Functionalization of Fe_3_O_4_ NPs: MNPs are dispersed in distilled water and stirred at room temperature. Copper oxide is added and stirred, then quantum dots are added, and the reaction is continued for 45 min. This solution is used in subsequent hydrogel formation. (2) Grafting of the gel: Dissolve SA and GM in distilled water, stirring magnetically. The functionalized Fe_3_O_4_ NPs are added, and the mixture is stirred. (3) Curing of the hydrogel product: After cooling to room temperature, the CaCl_2_ solution is slowly dispensed from a syringe held 15 cm above the mixture. The beads are stirred to ensure complete gelation, then washed with distilled water and dried, yielding GM-SA/Fe_3_O_4_@CuO@CQD hydrogel microspheres.

The grafting approach enables strong cross-linking between MNPs and the SA matrix, minimizing particle aggregation and leakage, thereby maintaining uniform distribution and enhancing MNPs in the matrix. However, this technology often involves more complex chemical processes and higher production costs, which may limit its application.

Each MSABH’s synthesis strategy has distinct advantages and limitations, underscoring the need for further research to address these challenges and develop more efficient preparation methods. Table 1 summarizes the concepts, benefits, and drawbacks of various MSABH fabrication techniques. In the subsequent section, recent advancements in the biomedical applications of MSABHs will be discussed, with a focus on targeted drug delivery, tissue engineering, and thermotherapy.

## 5. Biomedical Applications

Both environmental factors and the intrinsic properties of the polymer matrix strongly influence the behavior of MSABHs as externally responsive materials. A hallmark of magnetic materials is their ability to produce magnetically induced motion when subjected to magnetic field gradients. Depending on the frequency of the applied alternating magnetic field (AMF), MNPs can convert magnetic energy into other forms. At low-frequency AMF (<100 Hz), magnetic energy is changed into mechanical energy via dipole–dipole interactions [113]. These forces trigger structural modifications in MSABHs, providing stimulation to tissues or cells and provoking specific biological responses. At high AMF frequencies (100 kHz–1 MHz), the MNPs change magnetic energy into heat via 2 primary mechanisms: first, Néel relaxation, in which static MNPs’ internal magnetic moments overcome anisotropy; and second, Brownian relaxation, which arises from friction during MNPs’ motion. High-frequency magnetic fields (>1 MHz) primarily exert their effects through the magnetothermic and magnetic resonance effects. In tumor hyperthermia, MSABHs exposed to high-frequency alternating magnetic fields can rapidly generate heat, raising tumor tissue temperature to 42–45 °C, thereby effectively killing tumor cells. In addition, when MSABHs are used as contrast agents, they can significantly improve MRI resolution, providing strong support for early disease diagnosis [114]. The heat produced by MSABHs can be harnessed in diverse therapeutic areas, including drug delivery, hyperthermia treatment, tissue engineering, imaging, and microrobotics. Therefore, fine-tuning the polymeric and magnetic properties of MNPs, along with selecting appropriate external conditions, is critical to maximizing their efficacy in biomedical applications.

### 5.1. Stimuli-Responsive Drug Delivery Systems

Multiresponsive drug delivery systems represent a significant area of development in smart drug delivery. A multiresponsive delivery system is a smart drug carrier capable of eliciting reversible or synergistic responses to two or more external stimuli (such as pH, temperature, enzymes, light, magnetic fields, or redox conditions). Compared to single-responsive systems, multiresponsive designs enable more precise control over the spatiotemporal release of drugs, overcoming complex physiological barriers, enhancing therapeutic efficacy, and reducing adverse effects. SA, a natural polysaccharide derived from brown algae, has emerged as a key biomaterial in biomedical applications, particularly in the design of drug delivery systems [115]. Its notable advantages include biocompatibility, biodegradability, high drug loading and release efficiency, cell affinity, and absorption capacity [116]. Beyond their capacity for controlled drug release, SA-based systems are adaptable to multiple administration routes, including oral, intravenous, and intranasal. However, polysaccharide-based hydrogels may exhibit limited mechanical strength, thereby limiting their performance in drug delivery applications. The incorporation of MNPs into SA matrices not only enhances mechanical stability and functional performance but also improves magnetic responsiveness, enabling greater control over drug release. Combining SA with MNPs further broadens the applicability of MSABHs in drug delivery, allowing precise control of drug release at targeted sites and modulation of release kinetics. This targeted approach optimizes therapeutic outcomes while minimizing sudden fluctuations in plasma drug levels, thereby reducing the likelihood of adverse effects. Additional advantages of MSABHs-based drug delivery systems include improved patient compliance due to lower required doses, extended shelf life, and enhanced protection of therapeutic agents from environmental stressors such as extreme pH, heat, light, oxidative conditions, and chemical interactions. The material’s composition or structure can respond to these stimuli, facilitating controlled release of drugs at specific locations.

**Figure 2:**
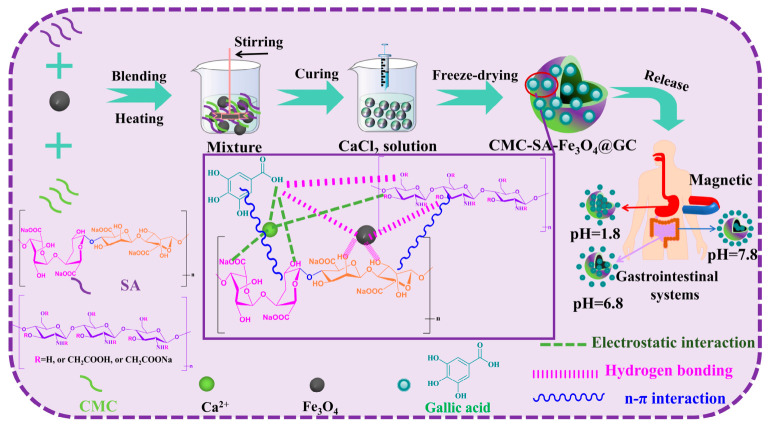
MSABHs are stimuli-responsive drug delivery systems for the controlled release of GA [117]. Adapted from an open access publication with permission, 2025, MDPI.

In a previous investigation, novel composite hydrogel beads (CMC-SA-Fe_3_O_4_@GA) that respond simultaneously to pH and magnetic field stimuli were developed using cost-effective_,_ biocompatible components: carboxymethyl chitosan (CMC), SA, and Fe_3_O_4_ NPs. These beads served as delivery systems for gallic acid (GA), as shown in Figure 2 [117]. The in vitro release studies indicated that these beads maintained remarkable stability under acidic conditions while achieving cumulative release in alkaline media, following the Ritger-Peppas kinetic model. Furthermore, exposure to an AMF significantly increased GA release. In a separate study, Zhang et al. [118] designed a hydrogel for osteosarcoma therapy by co-encapsulating Cu-Fe_3_O_4_ nanoenzymes (NCs) and artemisinin (AS) in situ, using SA and calcium ions. This hydrogel facilitates the sustained release of both NPs and AS within tumor tissues, leveraging the multi-enzymatic activity of NCs to promote the accumulation of reactive oxygen species (ROS). Carbon radicals (•C) produced through the Fe^2+^/Cu^2+^-AS reaction further enhance oxidative stress, resulting in tumor cell damage. At the same time, NCs trigger ferroptosis by depleting glutathione (GSH), thereby activating the GPX4 pathway, and induce copperptosis through intracellular copper overload, which activates the DLAT pathway, collectively boosting therapeutic effectiveness. In vitro assays demonstrated that the NCs-AS-SA hydrogel exhibits strong tumoricidal activity, while in vivo studies confirmed its capacity to effectively eradicate tumors with high biocompatibility, making it a promising strategy for osteosarcoma treatment.

MSABHs provide a good platform for developing intelligent drug delivery systems, with their outstanding performance arising from the combined effects of MNPs and SA. SA can cross-link under mild conditions to form hydrogels that efficiently encapsulate therapeutic agents and achieve controlled, pH-responsive release, for instance, targeted drug delivery in the slightly acidic environment of tumors. The incorporated superparamagnetic MNPs allow the hydrogel to respond to AMFs. When an AMF is applied, MSABHs can be directed and concentrated within specific diseased tissues, such as tumors, thereby markedly increasing local drug accumulation while minimizing systemic toxicity to healthy tissues and improving the therapeutic index.

### 5.2. Tissue Engineering Applications

Tissue engineering has emerged as a major advancement in regenerative medicine, offering novel strategies to repair or replace damaged tissues and organs by integrating principles from life sciences and engineering [119]. This approach involves generating biological substitutes or reconstructing tissues through in vitro or in vivo cultivation under controlled conditions, intending to restore, maintain, or enhance tissue functionality. As a rapidly evolving research area, it provides promising therapeutic avenues for a range of diseases. In recent years, polysaccharides have attracted considerable attention in the development of high-performance biomedical materials due to their advantageous characteristics, such as renewability and non-toxicity. Among these, SA has been widely employed in tissue engineering due to its mild gelation behavior and ease of chemical modification [120]. SA is especially valued for its good biocompatibility, which allows it to integrate seamlessly into biological systems while minimizing immune responses, a critical feature for tissue engineering applications [121]. However, SA also has limitations: its mechanical strength often falls short of natural tissues, particularly in applications requiring robust structural support (such as bone tissue engineering). This deficiency may compromise the durability and performance of SA-based constructs in dynamic physiological environments.

Furthermore, unmodified SA inherently lacks strong cell adhesion capabilities due to the absence of cell-binding sites. Applications requiring robust cell integration and tissue formation may necessitate further modification or the addition of other biomaterials [122]. This limitation makes it challenging to customize SA-based scaffolds for specific purposes while preserving their core properties [123]. Fortunately, MSABHs have emerged as highly suitable candidates for tissue engineering, offering easy processability into diverse geometries, along with favorable degradability, hydrophilicity, and tunable mechanical characteristics.

**Figure 3:**
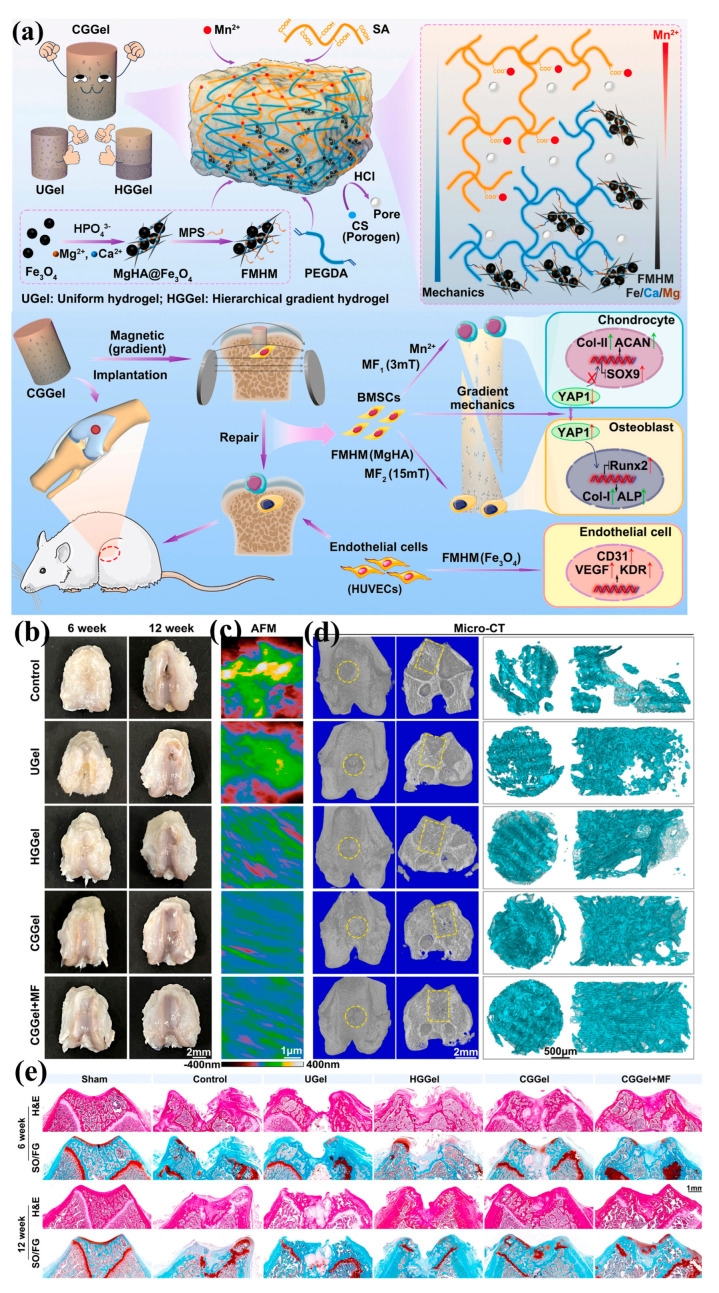
MSABHs in tissue engineering applications. (**a**) H&E-stained micrographs of tissue slices obtained from empty pouches, SPCL scaffolds, and magnetic scaffold groups. (**b**) Macroscopic views of osteochondral regeneration in the Control, UGel, HGGel, CGGgel, and CGGgel + MF groups at 6 and 12 weeks. (**c**) Surface morphology of the newly formed osteochondral tissue after 12 weeks. (**d**) Three-dimensional micro-CT reconstructions of the repaired osteochondral regions at week 12. (**e**) Histological sections showing H&E and SO/FG staining of the regenerated tissues at 6 and 12 weeks [124]. Adapted from an open access publication with permission, 2025, Elsevier.

Xu et al. [124] developed an innovative hydrogel scaffold incorporating both a continuous magnetic-mechanical gradient and a multifunctional metal element gradient to achieve full-thickness osteochondral regeneration, as illustrated in Figure 3a. The scaffold utilized a biomimetic extracellular matrix (ECM) framework composed of SA and polyethylene glycol diacrylate (PEGDA), while fully exploiting the pore-forming ability of chitosan (CS), the magnetic responsiveness of MNPs, and the bone–cartilage formation-promoting properties of manganese ions (Mn^2+^) and magnesium hydroxyapatite (MgHA). A gradient distribution of magnetic particles (Fe_3_O_4_ deposited on MgHA and grafted with γ-(methacryloyloxy)propyl trimethoxysilane, FMHM) was induced using a magnetic field, followed by secondary crosslinking and thermal curing, yielding a continuous magnetically responsive hydrogel. During fabrication, the magnetic field generated a pronounced gradient in net magnetism. Simultaneously, SA and Mn^2+^ exhibited gradient distributions driven by FMHM, the polymer network formed through PEGDA crosslinking with FMHM double bonds, and Mn^2+^ diffusion, ultimately producing counter-gradient distributions of Mn and Fe, as well as Mg and Ca. Continuous gradient hydrogels (CGGel) were implanted into rat cartilage–bone defects to repair tissues with a cartilage–subchondral bone gradient interface. The scaffold’s mechanical gradient likely promoted the synchronized growth of cartilage and subchondral bone by regulating the gradient nuclear localization and expression of the mechanosensitive factor Yes-related protein 1. Evaluation of cartilage–subchondral bone regeneration was conducted in vivo for CGGel, uniform hydrogel (UGel), and hierarchical gradient hydrogel (HGGel), combined with a gradient magnetic field (MF), as shown in Figure 3b–e. At week 6, the control and UGel groups displayed uneven cartilage surfaces with significant fibrosis, while HGGel showed partial improvement. The CGGel + MF and CGGel groups maintained smoother cartilage surfaces. By week 12, cracks persisted in both the control and UGel groups, and the HGG showed incomplete cartilage coverage. In contrast, CGGel and CGGel + MF fully repaired cartilage defects, with the CGGel + MF group achieving the smoothest surface. Atomic force microscopy (AFM) revealed progressively reduced cartilage surface roughness from control to UGel, CGGel, HGGel, and CGGel + MF groups, suggesting improved repair efficacy (Figure 3c). Micro-CT imaging demonstrated newly formed subchondral bone (Figure 3d), with week 6 showing large voids in control samples and limited bone in UGel. By week 12, control defects remained prominent, UGel showed moderate bone formation, and a histological analysis using H&E and SO/FG staining revealed incomplete hydrogel degradation at week 6 in most groups (Figure 3e); however, CGGel + MF showed accelerated degradation relative to CGGel, likely due to magnetic-field-enhanced cell growth and infiltration. By week 12, control samples retained defects, and UGel hydrogels remained in the cartilage area. Compared with granulation tissue in UGel and HGGel, CGGel and CGGel + MF exhibited more complete cartilage–bone interfaces, including proper defect filling, chondrocyte morphology, surface smoothness, cartilage thickness, and tear-line integrity. Histological scores (Wakitani and Seller) decreased progressively from control to CGGel + MF, reflecting superior regenerated tissue quality. Overall, CGGel with a continuous magnetomechanical gradient significantly improved integrated subchondral bone repair compared to uniform UGel and hierarchical HGGel scaffolds. Moreover, applying a gradient magnetic field further enhanced regenerative outcomes. This study highlights the potential of MSABHs for tissue and interface regeneration through the synergistic effects of multiple gradient metal elements, combined with stiffness and magnetic gradients.

In a separate study, Hia et al. [125] synthesized superparamagnetic iron oxide NPs (Sp) and coated them with a calcium phosphate layer to obtain distinctive flower-like microcluster morphologies (m-Sp). Subsequently, SA microbead hydrogels incorporating m-Sp (McSa@m-Sp) were fabricated via drop-casting. McSa@m-Sp demonstrated magnetic targeting capabilities, enhanced cross-linking efficiency, adjustable degradation rates, and strong antibacterial activity. Within the microsphere hydrogel, MC3T3-E1 cells exhibited significantly increased mineralization and alkaline phosphatase (ALP) activity, accompanied by elevated collagen production, as evidenced by alizarin red S (ARS) and Von Kossa staining. Immunocytochemistry and gene expression analyses further confirmed the osteogenic induction potential of McSa@m-Sp, with markedly higher expression of osteogenic markers including type I collagen, osteocalcin, RUNX-2, and osteopontin. Overall, MSABHs serve as a multifunctional platform for bone tissue regeneration, delivering bioactive substances, supporting cellular growth, and promoting osteogenesis to facilitate skeletal repair and reconstruction.

MSABHs combine biocompatibility and remote magnetic responsiveness, making them a highly promising smart biomaterial for tissue engineering applications. Their primary functions include constructing cellular scaffolds and facilitating magnetically guided tissue regeneration. Hydrogels formed by SA crosslinking provide a 3D environment that supports cell adhesion, proliferation, and differentiation. The embedded MNPs impart advanced functionality: AMFs enable precise control over scaffold morphology, mechanical properties, and the spatial distribution of growth factors, thereby directing cell alignment and growth, which are essential for the repair of anisotropic tissues such as tendons and nerves. However, prolonged exposure to magnetic fields may lead to cellular fatigue and damage. Long-term exposure to high-intensity magnetic fields can inhibit cell proliferation and induce apoptosis. Therefore, in the application of MSABHs, it is necessary to appropriately control the duration of magnetic field exposure to meet specific biomedical requirements and achieve optimal biological effects. In addition, this composite serves as a targeted delivery system, effectively concentrating adsorbed stem cells or bioactive factors at injury sites under magnetic-field guidance. This targeted approach significantly improves repair outcomes, offering an innovative and robust platform for regenerative treatment of tissues, including skin, cartilage, and bone.

### 5.3. Imaging

MRI is extensively recognized as an essential diagnostic modality in medical imaging. Moreover, MRI images are primarily categorized by relaxation mechanism: (A) T_1_-Weighted (longitudinal relaxation time) and (B) T_2_-Weighted (transverse relaxation time) images [126]. Gadolinium diethylenetriamine pentaacetate (Gd-DTPA), a paramagnetic complex, is commonly employed as a T_1_ contrast agent, whereas MNPs function effectively as T_2_ contrast agents [127]. This differentiation is particularly advantageous because, when combined with precise spatial and temporal control of magnetic field gradients, the enhanced magnetic field strength in MRI systems enhances imaging quality [128]. Additionally, MRI’s ability to generate high-contrast soft-tissue images enables real-time, interactive monitoring of cell localization, thereby verifying the efficiency of targeting strategies. Cells appear as low-density or dark regions in MRI images primarily due to the interaction between the magnetic field around each particle and the spin of paramagnetic molecules, which improves image contrast, especially in T_2_-weighted images containing MNPs [129]. However, exposed MNPs are susceptible to oxidation under environmental conditions and tend to aggregate due to their large SSA and strong intrinsic magnetic dipole interactions, resulting in rapid clearance by the reticuloendothelial system (RES) [130]. Therefore, surface functionalization strategies are essential to preserve MNPs’ intended application properties.

In this context, Lu et al. [131] developed a multifunctional therapeutic nanoplatform based on aldehyde-oxidized SA-stabilized Fe_3_O_4_ NPs, specifically engineered for T_2_-weighted MRI. Initially, SA oxide and Fe_3_O_4_ NPs were synthesized separately, then combined at a defined molar ratio to form a composite material. This composite was further coupled with the targeting peptide GE11 through a Schiff base reaction, followed by cisplatin loading via coordination complexation, yielding the GE11-CDDP-ASA@Fe_3_O_4_ complex for MRI applications. MRI imaging revealed that following intravenous injection of GE11-ASA@Fe_3_O_4_ and ASA@Fe_3_O_4_ into mice, both agents exhibited significant low-signal contrast enhancement at the HONE-1 tumor site. The complex preferentially accumulated in tumor tissue, with only the liver showing higher accumulation levels. Accumulation in the spleen, lungs, kidneys, and heart was relatively low, further demonstrating the high tumor-targeting efficiency of this nanoplatform in vivo.

**Figure 4:**
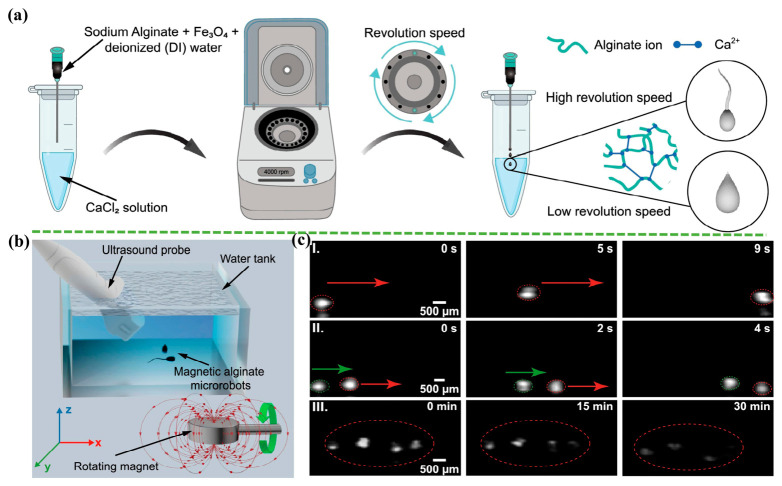
MSABHs for imaging applications. (**a**) Magnetic SA microrobots, including teardrop-shaped and tadpole-shaped microrobots, were fabricated using centrifugal flow and cross-linking methods involving divalent calcium ions. (**b**) Schematic diagram of the magnetic-driven micro-robot ultrasonic imaging experimental setup. (**c**) Time-lapse ultrasonic images reveal: (**I**) Rolling motion of a single micro-robot, (**II**) Rolling motion of two micro-robots, and (**III**) Degradation process of multiple micro-robots [132]. Adapted from an open access publication with permission, 2024, Elsevier.

In ultrasound imaging, MSABHs markedly improve contrast through two main mechanisms. First, the embedded MNPs function as acoustic impedance modulators, enhancing echo differences between materials and surrounding tissues. Second, the SA matrix forms a microbubble-rich porous network that strongly reflects ultrasound, thereby improving lesion visualization. Under the guidance of an AMF, the composite can also achieve targeted accumulation at specific sites, such as tumor regions, enabling precise molecular imaging. Moreover, MSABHs can be biomimetically engineered as microrobots mimicking natural organisms, including tadpoles, spermatozoa, and fish. Their propulsion is achieved by replicating undulating tail motions, as observed via ultrasound imaging. For instance, Wang et al. [132] fabricated size-adjustable droplet-shaped and tadpole-shaped MSABHs microrobots by driving fluid through a controllable centrifugal force, and visualized simulated fluid dynamics around the microrobots during rolling and stick-slip motions (Figure 4a). During rolling, the teardrop-shaped microrobot exhibited increasing flow velocities at both the front and rear regions. In contrast, the tadpole-shaped microrobot showed maximum flow velocity at the tail and reduced velocities around the head. This suggested that higher tail velocities facilitated forward motion during stick-slip behavior. The study also utilized an ultrasound imaging system to track the microrobots’ positions by exploiting the acoustic impedance mismatch between the microrobots and the surrounding fluid. The experimental setup included a rotating permanent magnet and the ultrasound system (Figure 4b). Microrobots were placed in a water tank, with the ultrasonic probe positioned at a specific angle to monitor the motion and degradation of the MSABHs microrobots. Both teardrop- and tadpole-shaped microrobots demonstrated rolling motion under the rotating magnet. Delayed rolling sequences of single and paired teardrop-shaped microrobots are shown in Figure 4c(I) and c(II), respectively. Figure 4c(III) depicts the degradation process of multiple microrobots, captured via ultrasound. Over time, imaging signal intensity decreased, indicating ongoing hydrogel degradation, while faint signals persisted for up to 20 min due to MNPs remaining on the substrate after SA hydrogel breakdown.

In MRI applications, MSABHs serve primarily as high-performance T_2_-weighted contrast agents. The embedded MNPs strongly modify local magnetic fields, accelerating proton relaxation and thereby significantly enhancing imaging contrast between abnormal regions, such as tumors, and surrounding normal tissues. This capability enables more accurate localization and diagnosis. Additionally, due to SA’s high modifiability, targeting molecules can be conjugated to the surface, promoting active accumulation of the complex within specific diseased cells and improving the sensitivity of molecular imaging. In ultrasound imaging, MSABHs act as innovative contrast agents. Their pronounced acoustic impedance mismatch with human tissue efficiently scatters ultrasound waves, amplifying echo signals to clearly outline the structure of blood vessels and specific tissues. The MNPs within MSABHs also provide magnetic targeting capabilities. Application of an AMF allows the contrast agent to be actively guided and concentrated at target sites, such as tumors, enhancing local signal-to-noise ratio and detection sensitivity. By combining ultrasound imaging with magnetic targeting, MSABHs offer a safe and effective platform for high-precision, high-sensitivity disease diagnosis, particularly useful for early tumor detection and assessment.

### 5.4. Hyperthermia

Hyperthermia has demonstrated significant potential in cancer therapy due to its capacity to selectively target tumor cells. Various technologies are currently utilized to induce hyperthermia, including induction heating, capacitive heating, and microwave heating [133]. Unlike conventional treatments such as radiotherapy and chemotherapy, magnetothermolysis exploits cancer cells’ characteristic rapid DNA replication. This strategy not only minimizes toxic effects on healthy tissues but also helps overcome tumor resistance arising from genetic mutations [134]. A key challenge for clinical implementation of hyperthermia is achieving precise thermal targeting of tumors while avoiding damage to adjacent healthy tissues [135]. MSABHs are generally composed of biocompatible SA combined with MNPs (e.g., Fe_3_O_4_ NPs) that exhibit magnetothermal properties. SA provides a stable hydrogel carrier through its inherent gelation, biodegradability, and mild cross-linking conditions, thereby preventing in vivo aggregation of MNPs and enabling molecular functionalization. When exposed to an AMF, the encapsulated MNPs efficiently convert magnetic energy into heat, acting as localized microheaters for targeted tumor treatment.

**Figure 5:**
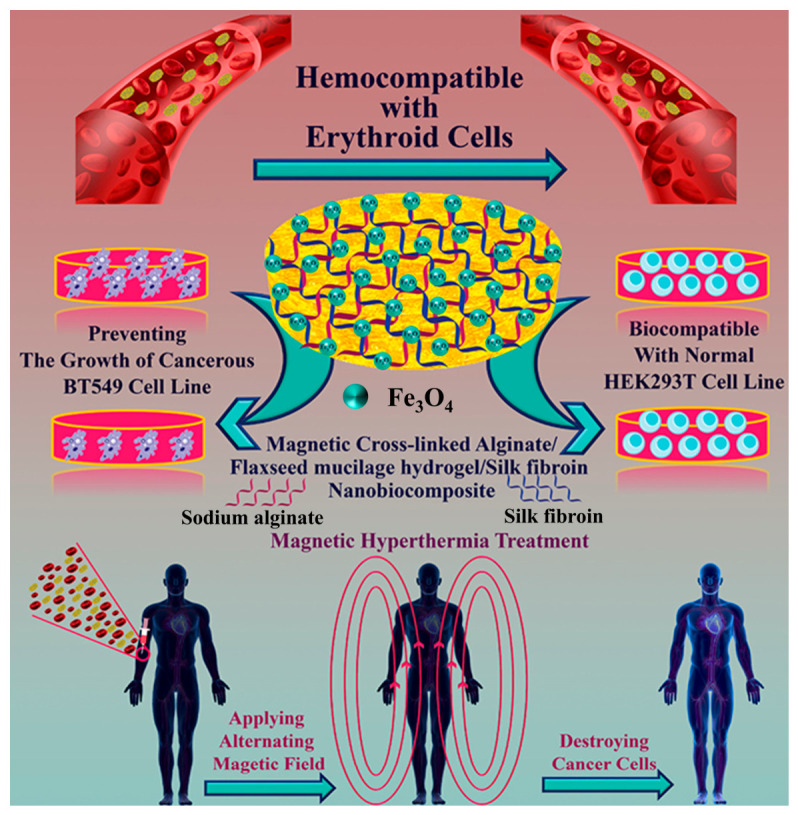
Magnetic cross-linked alginate-biobased nanocomposite with hyperthermia activities [136]. Adapted from an open access publication with permission, 2024, Elsevier.

In prior research, Radinekiyan et al. [136] prepared a cross-linked SA/flaxseed mucilage hydrogel/silk fibroin nanobiocomposite and applied it to magnetothermotherapy. Biological experiments demonstrated that the survival rates of normal HEK293T cells and breast cancer BT549 cells, respectively, validated the biocompatibility and anticancer properties of this composite. A hemolysis effect of less than 5% indicates its hemocompatibility. Furthermore, a high specific absorption rate was achieved at a concentration of 1 mg/mL (Figure 5). In a separate study, Sun et al. [137] engineered a smart composite scaffold capable of combining magnetic-controlled hyperthermia with chemotherapy by integrating drug-loaded thermosensitive liposomes, MNPs, and the biodegradable polymer SA. EMF not only elevates the matrix temperature during magnetothermic therapy but also triggers the release of DOX for chemotherapy. Both in vitro and in vivo experiments have confirmed the synergistic anticancer effects of this composite matrix. Under the influence of an electromagnetic field, the composite matrix effectively eliminates breast cancer cells. Furthermore, the matrix promotes the proliferation and adipogenic differentiation of mesenchymal stem cells to reconstruct adipose tissue following anticancer treatment. Regeneration experiments indicate that this composite scaffold effectively maintains its structural integrity and promotes the infiltration and proliferation of normal cells within the scaffold. With its multifunctional properties, this composite scaffold offers a highly promising new platform for the effective treatment of breast cancer.

MSABHs provide an effective combination of magnetic targeting and thermally controlled drug release for tumor hyperthermia. Under the guidance of an external static magnetic field, the composite can selectively accumulate in tumor tissues. Subsequent exposure to an AMF allows the embedded MNPs (e.g., Fe_3_O_4_) to efficiently convert magnetic energy into heat, raising the local temperature at the lesion site to selectively ablate tumor cells. Importantly, the three-dimensional network of SA functions not only as a stable carrier but also as a platform for loading chemotherapeutic drugs, creating a synergistic therapeutic system. Upon local temperature elevation, the swelling behavior of SA changes, triggering intelligent drug release and enabling a coordinated enhancement of magnetic targeting, hyperthermia, and chemotherapy. With their precision, controllability, and high efficacy, MSABHs hold great promise for clinical translation.

### 5.5. Magnetic-Actuated Miniature Robots

In the era of precision medicine, conventional drug delivery approaches are increasingly insufficient for managing complex diseases due to limitations such as systemic side effects, drug resistance, and lack of targeting [138]. As a result, micro-robot technology capable of delivering therapeutic agents directly to diseased sites under external field control has gained prominence. Owing to their miniaturized size, multifunctionality, and high maneuverability, wireless micro-robots are emerging as practical tools in minimally invasive biomedical applications [139]. Additionally, these micro-robots can operate in restricted or hard-to-reach anatomical spaces, assisting surgeons in performing precise interventions [140]. MSABs have become an ideal material platform for biomedical microrobots because of their unique properties, including good biocompatibility, non-toxicity, natural biodegradability, and magnetic responsiveness, which enable precise remote actuation, positioning, and tracking via AMFs. On one hand, MSABHs facilitate precision-targeted therapy. Guided by AMFs, drug-loaded MSABHs navigate complex biological environments like “micro-submarines,” reaching tumors or sites of inflammation. They subsequently degrade and release drugs in response to acidic or enzymatic stimuli within the tumor microenvironment, significantly improving therapeutic outcomes while minimizing damage to healthy tissues. On the other hand, they offer substantial potential in minimally invasive surgery and interventional procedures, functioning as micro-manipulation tools for clearing vascular emboli, delivering cells, or performing localized microsurgical tasks.

**Figure 6:**
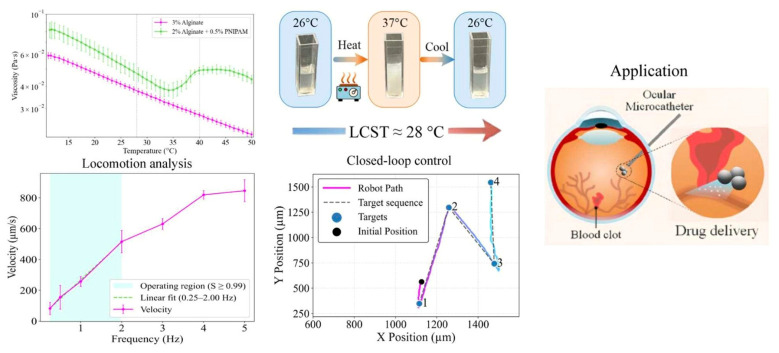
MSABHs for magnetic-actuated miniature robots [141]. Adapted from an open access publication with permission, 2026, Elsevier.

In this study, Khedewy et al. [141] prepared thermosensitive hydrogel microrobots (with an average diameter of approximately 194 µm) based on alginate and poly(N-isopropylacrylamide) (PNIPAM), uniformly loaded with iron oxide nanoparticles to enable wireless magnetic propulsion in Figure 6. This composite hydrogel exhibits a reversible phase transition within a lower critical solution temperature (LCST) range of 28–40 °C, resulting in temperature-dependent changes in its viscosity and optical properties. Under a 10 mT rotating magnetic field, the microrobots exhibit stable rolling motion, with velocity linearly proportional to frequency over a slip ratio close to 1. Under open-loop, closed-loop, and collaborative swarm control, their translational velocity remained between 112 and 169 µm/s. Swarm-level driving resulted in an average heading error of approximately 12.4° and a lateral error of approximately 17 µm; in contrast, closed-loop control achieved convergence to sequential targets with a steady-state error of approximately 45 µm. As a proof-of-concept for non-planar environments, the microbot successfully climbed a PDMS surface mimicking the curvature of an eyeball, with a slope of up to approximately 50°. These results establish alginate/PNIPAM hydrogels as a viable material platform for responsive micro-robotic systems, highlighting their potential value in minimally invasive biomedical applications, including targeted therapy, ophthalmic microsurgery, and, in particular, future applications in military-related trauma (such as vitreous hemorrhage rehabilitation).

In a related study, Wu et al. [142] developed a hollow magnetic soft robot (HMSR) by combining SA ion bonding with polyethylene glycol diacrylate (PEGDA) polymerization for drug loading. A magnetic navigation platform was utilized to achieve precise drug delivery to lesions within a vascular model. Due to its flexible fiber structure and low stiffness, the HMSR can autonomously navigate target blood vessels and be fully retrieved through a surgical incision. Under magnetic guidance, the drug-loaded HMSR precisely reached the lesion entrance, and the release of rhodamine B (RB) was monitored using fluorescence microscopy. The drug release profile showed a rapid initial burst within 30 min, followed by sustained release over a 60 min maintenance phase, making it particularly suitable for thrombolysis or chemotherapy applications. To compare drug release between single-network and dual-network HMSRs, RB was loaded into a single-network SA-based HMSR. Fluorescence intensity (FI) at the lesion entry increased rapidly during the first 30 min but decreased by 90 min. Meanwhile, FI within the single-network HMSR declined linearly from 1 to 90 min. This demonstrates that the dual-network HMSR (SA + PEGDA) effectively delays drug release by creating a denser diffusion barrier.

MSABHs-based micro-robots provide precise control and effective coordination across the entire diagnostic, therapeutic, and tissue-repair process by combining multiple functions within a single system. They not only embody a technological breakthrough but also serve as a crucial platform for next-generation medical applications, offering novel opportunities to tackle major diseases.

## 6. The Toxicity of MSABHs

MSABHs exhibit significant promise in biomedical applications, including magnetothermolysis, tissue engineering, and targeted drug delivery, owing to their good biocompatibility, degradability, and magnetic responsiveness. Nevertheless, a major barrier to their clinical translation is the need for a thorough toxicological evaluation. The potential toxicity of MSABHs may stem from their individual components, particularly SA and MNPs. Although SA is generally considered safe, factors such as its source, molecular weight, purity, degradation byproducts, and chemical modifications can trigger immune responses or lead to long-term accumulation [143]. MNPs, on the other hand, may induce ROS formation via Fenton reactions, leading to oxidative stress, cellular damage, inflammation, and possible alterations in gene expression [144]. Multiple factors influence MSABHS toxicity, including concentration and dosage, the chemical composition of the SA matrix and MNPs, synthesis techniques, particle geometry, and size.

Additionally, tissue or organ type, exposure duration, intracellular localization, and interactions with cellular components such as the cytoskeleton play critical roles. Other considerations, including administration route, pharmacokinetics, and biodistribution, collectively determine the overall toxicological profile. Toxicity assessment of MSABHs generally involves both in vitro and in vivo approaches. In vitro studies typically involve incubating MSABHs in culture media, applying the conditioned medium to cells, and evaluating toxicity by assessing changes in cell morphology, proliferation, and functional activity. In vivo evaluations in animal models are more complex, focusing on abnormal physiological responses, mortality following dosing, and long-term studies assessing distribution, metabolism, and accumulation within the body.

In their study, Foroutan et al. [145] fabricated Fe_3_O_4_ NPs (NPs) in situ on a hydroxyapatite (HAp) substrate. Subsequently, they incorporated the HAp-Fe_3_O_4_ composite into an SA biopolymer for targeted delivery of the anticancer drug 5-fluorouracil (5-FU). Therapeutic efficacy and biocompatibility were assessed utilizing HT-29 cells exposed to free 5-FU, blank formulations, and 5-FU-loaded SA/HAp-Fe_3_O_4_ nanocomposites. As illustrated in Figure S10a(i), the blank nanocomposite demonstrated negligible cytotoxicity across a 5–60 mg/mL concentration range after 24 and 48 h. However, the concentration gradient differs from that used for the drug-loaded formulation. In contrast, Figure S10a(ii) showed that SA/HAp-Fe_3_O_4_ loaded with 5-FU (4–48 μg/mL) displayed lower cytotoxicity at 48 h compared to free 5-FU. These MTT assay results indicate that SA/HAp/Fe_3_O_4_ exhibits good biocompatibility, while 5-FU-loaded SA/HAp-Fe_3_O_4_ demonstrates substantial cytotoxicity against HT-29 human colon cancer cells. Similarly, Kumar and Gopinath [146] developed a three-dimensional in vitro tumor model using porous microspheres composed of SA and SA/silk fibroin (SA/SF). They further evaluated magnetic-induced apoptosis by incorporating MNPs into the SA/SF microspheres. The in vitro cytotoxicity of SA alone and SA-SF (1%, 2%, 3%, 4%, and 5%) against normal lung embryonic cells (L132) was examined in Figure S10b(i). After 72 h, lyophilized SA microspheres at high concentrations (5%) exhibited some cytotoxicity. In contrast, SA-SF microspheres showed no toxicity compared with controls, likely because silk proteins mimic extracellular matrix components and promote cell adhesion and proliferation. Figure S10b(ii) demonstrated cytotoxicity of SA/SF microspheres with or without MNPs against A549 lung cancer epithelial cells. High MNP concentrations can generate ROS and oxidative stress, causing cell death; however, encapsulation within SA/SF microspheres prevented direct cell contact, thereby avoiding ROS-mediated toxicity. Cells within SA/SF microspheres containing MNPs were exposed to static magnetic fields of low (~38 mT) and high (~95 mT) intensity for 24 h, and cellular responses were evaluated using AO/EB dual staining (Figure S10b(iii–vii)). AO stains intact nuclei of viable cells green when bound to double-stranded DNA, and red when bound to single-stranded DNA or RNA. EB penetrates ruptured membranes, intercalates into DNA, and emits orange fluorescence. During early apoptosis, intact membranes retain green granular nuclei indicative of nuclear fragmentation, whereas in late apoptosis, EB entry allows nuclei to become orange. Untreated 2D-cultured A549 cells showed green fluorescence, confirming viability (Figure S10b(iv)), while cells in SA/SF microspheres loaded with SPIONs remained green even without magnetic field exposure (Figure S10b(v)). Low-field (~38 mT) treatment induced early (yellow) and late (orange) apoptosis in cells embedded in porous magnetic beads (Figure S10b(vi)), whereas high-field (~95 mT) exposure led to necrosis (red) and cell death (Figure S10b(vii)). These investigations mainly addressed in vitro toxicity profiles.

In vivo toxicity assessments of MSABHs have also attracted considerable attention. Elderdery et al. [147] synthesized α-Fe_2_O_3_-SA-eugenol nanocomposites (FSE NCs) and investigated their biological safety using a zebrafish model. The study evaluated anticancer efficacy alongside hematological parameters, liver enzyme levels, and histopathological features (Figure S10c). After 7 days of exposure to varying FSE NC concentrations (25, 50, and 100 mg/L), Figure S10c(i–iii) demonstrated that ALT, AST, and ALP levels were significantly lower in treated groups compared to controls (*p* < 0.0001), indicating non-toxicity. Hematological analysis (Figure S10c(iv–vi)) revealed that red and white blood cell counts in treated zebrafish were comparable to those of the control group. In the differential white blood cell assessment, lymphocytes, monocytes, neutrophils, eosinophils, and basophils all displayed significant reductions (*p* < 0.0001), confirming the absence of hematotoxic effects. Furthermore, hematoxylin–eosin-stained liver tissue images collected after 7 days of treatment showed intact structural integrity with no notable abnormalities, highlighting the biocompatibility of FSE NCs (black arrows indicate hepatocytes in Figure S10c(vii)).

## 7. Conclusions and Future Perspectives

In summary, this review systematically demonstrates that magnetic nanoparticles (MNPs) are the key factors in regulating the size, morphology, and magnetic domain distribution of magnetic alginate hydrogels (MSABHs) and that the combination of alginate (SA) with different types of MNPs has been proven to be an effective strategy for constructing multifunctional magnetic composites. By comparing synthetic approaches such as in situ coprecipitation, graft modification, and physical mixing, it was found that the interfacial bonding strength and uniformity of MNP distribution directly determine the synergistic performance of the hydrogels—namely, the combination of SA’s biocompatibility and pH-responsive gelation with the superparamagnetism and magnetothermic effects of MNPs. This enables integrated functions such as magnetically controlled release, thermoresponsiveness, targeted delivery, mechanical reinforcement, biocompatibility, and rapid separation, thereby supporting their applications in drug delivery, tissue engineering, imaging, hyperthermia, and micro-robotics. However, current research faces specific technical bottlenecks: regarding magnetic responsiveness, most MSABHs require magnetic fields exceeding 0.5 T for effective actuation, exhibiting insufficient low-field sensitivity; moreover, MNP aggregation weakens magnetic anisotropy, leading to response hysteresis. Regarding translation barriers, there is a lack of systematic pharmacokinetic/toxicokinetic data, particularly regarding the long-term elution behavior of MNPs from the SA network and the biosafety of degradation products; existing evaluations are largely limited to short-term in vitro models; In terms of practical application, it is difficult to ensure consistency in magnetic performance across batches during large-scale production; there is a trade-off between mechanical strength and injectability/printability; and cost–benefit analyses are lacking. Distinguishing this review from existing literature, its original contribution lies in proposing, for the first time, an analytical framework that integrates “synthesis methods–interface interactions–synergistic performance–application constraints,” revealing how coordination cross-linking and electrostatic adsorption between MNPs and SA determine magnetic response efficiency and biofunctional output. Based on this, we identify key performance shortcomings and translation barriers, providing a perspective grounded in theoretical principles and concrete technical guidance for designing next-generation high-performance MSABHs and evaluating their clinical feasibility.

However, to facilitate broader clinical translation of MSABHs, several challenges remain to be overcome. The following highlights the primary directions for their future development in biomedical applications:

**1. Drug Delivery Systems: Targeting and Smart Diagnosis–Therapy Integration.** Magnetic fields can direct drug-loaded MSABHs to accumulate precisely at tumor sites, minimizing systemic toxicity while enhancing therapeutic effects. Future strategies may combine alternating and static magnetic fields to improve tissue penetration and drug anchoring. Moreover, the inherent pH sensitivity of MSABHs can be further optimized through molecular design to respond simultaneously to multiple stimuli (magnetic fields, enzymes, redox conditions), enabling programmed and multi-stimulus drug release. Real-time imaging tools, such as MRI, enable continuous monitoring of drug distribution and release, integrating diagnosis and therapy to dynamically adjust treatment regimens.

2. **Tissue Engineering: From Static to Dynamic Bionic Constructs.** Co-encapsulation of MNPs with seed cells in SA-based hydrogels enables spatially directed alignment and 3D ordered assembly under strong magnetic fields in vitro. This approach overcomes the structural constraints of traditional scaffolds, allowing fabrication of tissues with anisotropic architectures, including muscle, tendon, and organoid-like structures. In vivo, applying an AMF to MSABHS scaffolds generates subtle magnetomechanical stresses that mimic natural biomechanical forces, promoting stem cell differentiation along osteogenic and chondrogenic pathways and accelerating functional tissue regeneration.

3. **Hyperthermia and Synergistic Therapy: Toward Precision Platforms.** As magnetothermy and synergistic treatment strategies move toward clinical translation, a series of key bottlenecks still need to be overcome: there is currently no standardized evaluation system for the reproducibility and quality control of magnetic materials from different batches, and there is an urgent need to establish unified procedures for material characterization and verification of thermal effects; conventional sterilization methods may alter material properties, necessitating the development of low-temperature sterilization processes suitable for magnetic nanomaterials, as well as standards for rapid endotoxin detection and removal; Magnetothermal materials accumulate in organs such as the liver and spleen in vivo, and their long-term toxicity and functional effects remain unclear; systematic pharmacokinetic and toxicokinetic studies should be conducted; there is a lack of evidence regarding the clearance pathways of non-degradable materials and the metabolic fate of degradation products from degradable materials (such as iron-based ones), necessitating the design of systems with well-defined clearance mechanisms; There are currently no clear clinical safety thresholds for the potential risks to normal human tissues posed by the frequency, amplitude, and exposure duration of alternating magnetic fields. Risk-based exposure guidelines must be established in conjunction with treatment depth and thermal field distribution. Furthermore, while combined magnetothermal and immunotherapy holds potential for synergistic effects, the impact of magnetic fields on immune cell function and cytokine levels requires further evaluation. Future studies should validate the safety window and long-term effects of combined regimens in preclinical models.

4. **Medical Imaging and Diagnosis: From Single-Modal to Multimodal Probes.** MSABHs can be engineered for simultaneous use in MRI (soft tissue resolution), CT (spatial resolution), photoacoustic imaging (penetration depth and sensitivity), and other modalities. These multimodal probes provide complementary diagnostic information for accurate lesion localization and characterization. They also act as therapeutic agents, allowing clinicians to identify lesions in real time and trigger drug release or hyperthermia on demand, achieving seamless integration from diagnosis to treatment.

5. **Micro/Nano-Robots: Remote, Precise In-Body Operations.** There are batch-to-batch variations in the motion performance and magnetic responsiveness of micro- and nano-robots, necessitating the development of high-precision, high-throughput manufacturing processes and performance quality control standards. The complex surface structures of these robots can easily lead to microbial adhesion and endotoxin residue; traditional cleaning and sterilization methods struggle to remove these contaminants thoroughly without compromising functionality. Therefore, sterile fabrication and terminal sterilization protocols suitable for micro- and nano-devices should be developed. After navigating through blood vessels, robots distribute to organs such as the liver, lungs, and kidneys. Due to their minute scale, it is difficult to track their long-term accumulation effects; high-resolution imaging and isotope-labeling technologies are needed to map their spatiotemporal distribution. Future trends require robots to be fully biodegradable, with degradation products that are non-toxic and excretable through normal metabolic pathways. However, current knowledge regarding the alignment of degradation rates with mission durations and the safety of degradation intermediates is limited. Enzyme- or pH-responsive degradation mechanisms should be designed, and the final elimination pathways (e.g., urine, feces, or CO_2_) should be clearly defined. Prolonged and multi-regional exposure to alternating or rotating magnetic fields required for robotic propulsion may induce eddy-current heating in tissues or neuromuscular stimulation. Currently, there are no safety standards for AMF regarding dynamic swarm movements. It is recommended to establish dose–response models by integrating magnetic-field spatial distributions with drive modes. Finally, closed-loop systems integrating imaging feedback with robotic control are still in the proof-of-concept stage. Imaging accuracy, magnetic-field compatibility, and the safety of long-term tracking must be validated in large-animal models.

6. **In Vitro and In Vivo Toxicity of MSABHs.** The clinical translation of MSABHs requires overcoming toxicity-related challenges, with risks spanning multiple dimensions, including oxidative stress, cytotoxicity, prolonged retention, in vivo distribution, and metabolic fate. Surface modification is a key mitigation strategy: PEGylation can reduce protein adsorption and immunogenicity, while coatings such as chitosan and hyaluronic acid can enhance cell compatibility; the development of biodegradable magnetic components, such as iron oxide nanoparticles doped with biocompatible metals, can be gradually cleared via the reticuloendothelial system, thereby avoiding the risk of long-term accumulation. However, a gap remains between in vitro screening and the translation of results to in vivo applications. Standardized in vivo-in vitro-cell (IVIC) models, combined with organ-on-a-chip technology and pharmacokinetic simulations, are key to accurately predicting long-term biosafety. Only through an in-depth analysis of the mechanisms by which material properties interact with biological systems can we advance the safe application of low-toxicity, high-efficiency MSABH platforms for targeted drug delivery, implantable devices, and diagnostic imaging.

7. **In-depth evaluation of MSABHs’ standardization, repeatability, and regulatory approval.** Although magnetic sodium alginate-based hydrogels (MSABHs) show great promise for applications in drug delivery, tissue engineering, and other fields, their clinical translation still faces multiple bottlenecks related to standardization, reproducibility, and regulatory approval: there are no unified standards for raw material parameters during the preparation process, and industry-wide discrepancies exist regarding characterization methods and evaluation criteria, leading to fluctuations in product performance and making it difficult to compare research results across studies; Minor fluctuations in preparation processes and the complexity of in vivo biological systems further exacerbate uncertainties regarding product performance and clinical efficacy; the existing regulatory framework struggles to accommodate their dual nature as material carriers with therapeutic functions, with a lack of safety and efficacy evaluation standards and an unclear approval pathway. Moving forward, it will be necessary for the government to take the lead in establishing unified standards, for research institutions to deepen mechanistic studies, and for companies to strengthen quality control. Concurrently, cross-disciplinary collaboration and the incorporation of international best practices must be enhanced to construct a regulatory framework tailored to the characteristics of MSABHs. Only by promoting deep integration among industry, academia, research, and clinical practice can these bottlenecks be overcome and accelerate the translation of MSABHs from the laboratory to clinical applications.

In summary, future MSABHs are evolving toward highly intelligent, integrated, precise, and personalized applications that bridge materials science, nanotechnology, imaging, and clinical medicine, thus promising transformative advances in disease diagnosis and therapy.

## Figures and Tables

**Table 1:** Comparison of the disadvantages and advantages of different MSABH preparation methods.

Methods	Concept	Advantages	Disadvantages	Scalability Analysis
Self-assembly	Physical mixing of pre-synthesized MNPs with SA via intermolecular forces.	Mild conditions; simple operation; high biocompatibility; controllable MNPs properties.	Poor bonding/stability; low magnetic content; limited functional modification	High scalability: easy process amplification, low cost, suitable for continuous large-scale production
In situ co-precipitation	Direct addition of iron salts (Fe^2+^/Fe^3+^) to the SA solution enables the in situ formation of MSABHs within the polymer network under alkaline conditions.	Evenly distributed MNPs; High magnetic content; One-pot synthesis; Strong binding force; Low cost.	Uneven particle size; Alkaline environment; Biocompatibility risk; Polymer degradation risk.	Medium scalability: feasible amplification with optimized pH/temperature control, suitable for semi-continuous production
Grafting	MSABHs are obtained by covalently linking the molecular chains of SA to functional groups on the surface of MNPs (such as -COOH, -OH) via chemical bonds, or by modifying the surface of MNPs to undergo specific chemical reactions with SA.	Good stability; Highly functionalized; Good MNPs dispersion; Controllable structure.	Complex process; harsh conditions; high cost; potential toxicity.	Low scalability: multi-step complexity, strict condition control, suitable for small-batch laboratory preparation

## Data Availability

The original contributions presented in this study are included in the article and the Supplementary Materials.

## Supplementary Materials

The following supporting information can be downloaded at: https://www.mdpi.com/article/10.3390/gels12060508/s1, Figure S1: Scientific literature trend chart for the keyword “magnetic SA biomedical” retrieved via Google Scholar, with data available through 31 December 2025; Figure S2: (a) Structural formulas and the lowest-energy conformers of GG, MM, and GM units generated with AVOGADRO and optimized using the GAFF force field. (b) Isomeric regions of the ^1^H NMR spectra for alginate samples with varying M-block content, with peak assignments following the descriptions of Grasdalen [52]. Copyright 1983, Elsevier. (c) Snapshots of alginate chains (Nm = 30) in SA solution with alternating G/M monomer sequence GMGM, GMM, and GGM at different SA concentrations of Nc = 10 and 17. C. Reactive carboxy groups of SA [60]. Copyright 2016, ACS Publications; Figure S3: (a) SA gelation by calcium, the “egg-box” model [78]. Copyright 2020, Elsevier. (b) Alginate ionic gelation includes internal and external gelation [80]. Copyright 2022, Elsevier; Figure S4: The point and mechanism of chemical modification [93]. Copyright 2024, Elsevier; Figure S5: Fe_2_O_3_ fundamental crystal structures graphical representations: (a) α-Fe_2_O_3_, (b) β-Fe_2_O_3_, (c) γ-Fe_2_O_3_, (d) ε-Fe_2_O_3_ [101]. Copyright 2011, ACS publication; Figure S6: (a) Crystal structure of magnetite (Fe_3_O_4_) phase [102]. Copyright 2015, IOP Publishing; (b) Unit cell structure of magnetic spinel ferrite [103]. Copyright 2024, Elsevier; Figure S7: (a) Schematic representation of the preparation of magnetic SA microcarriers [106]. Copyright 2025, Elsevier. (b) Schematic representation of the fabrication of magnetically graded materials. (1) A ferrofluid composed of PAA-stabilized MNPs served as the starting material; (2) Magnetic inks were formulated with ferrofluid contents of 0%, 10%, and 20% (*w*/*w*); (3) These inks were then used for multimaterial 3D printing to construct magnetic structures; (4) The resulting patterned hydrogels exhibited distinct magnetic responses, enabling motions such as rolling (a), jumping (b), and bending (c) [107]. Copyright 2022, Elsevier.; Figure S8: (a) Schematic representation of the fabrication process and evaluation of the synergistic potential of SA-Trp-Fe_3_O_4_ [109]. Copyright 2025, Elsevier. (b) Preparation of SA-TA hydrogel/SF/Fe_3_O_4_ magnetic nanocomposites [110]. Copyright 2023, Elsevier; Figure S9: (a) Synthesis of SA/β-CD/Fe_3_O_4_@Ag MHBs [111]. Copyright 2025, Elsevier; (b) Synthesis of GM-SA/Fe_3_O_4_@CuO@CQD [112]. Copyright 2025, Elsevier; Figure S10: Toxicological evaluation of MSABHs. (a) (i) Cell viability of SA/HAp–Fe_3_O_4_ after 24 and 48 h (*p* < 0.05); (ii) Viability of cells exposed to 5-FU alone and to 5-FU-loaded SA/HAp–Fe_3_O_4_ at equivalent drug concentrations for 48 h (*p* < 0.05) [145]. Copyright 2025, Elsevier. (b) (i) Cytocompatibility of pure SA beads (1–5%) and silk-fibroin–incorporated SA beads (SA/SF, 1–5%); (ii) Viability analysis of bare MNPs (0.2–1.0 mg) and SF-containing SA beads (3% SA, lyophilized) loaded with MNPs at 2–10 mg; (iii) Illustration of the static magnetic field applied to cells cultured on MNP-embedded SA/SF porous beads; Live/dead staining of A549 cells: (iv) untreated control in standard culture plates; (v) cells within MNP-loaded porous beads without magnetic stimulation; (vi) cells exposed to a low static magnetic field (38 mT); (vii) cells subjected to a high static magnetic field (95 mT). Scale bar = 200 μm. Early apoptosis (EA) indicated by yellow dashed circles; late apoptosis (LA) by orange dashed circles; dead cells by red dashed circles [146]. Copyright 2023, Elsevier. (c) *In vivo* toxicology of FSE NCs. The results of liver enzyme AST(i), ALT (ii), and ALP (iii) in zebrafish blood were studied using iron oxide-SA nanocomposites supplemented with varying doses of eugenol (e.g., 25, 50, and 100 mg/L); (iv–vi) A study was conducted on adult zebrafish blood using iron oxide-SA nanocomposites supplemented with different doses of eugenol (e.g., 25, 50, and 100 mg/L); Analysis of the histopathological effects of eugenol in the liver tissue of adult zebrafish in FSE NCs [147]. Copyright 2024, Elsevier.
