# Supplementary material for: Recent Advancements in Sodium Alginate-Based Hydrogels Combined with Magnetic Nanoparticles for Biological Applications: A Review
Source: Gels. 2026 Jun 8;12(6):508. doi: 10.3390/gels12060508 (PMC13297855; doi:10.3390/gels12060508)
Supplement: Supplementary file 1 [file gels-12-00508-s001.zip › gels-4347105-supplementary.pdf]

Supplementary Materials

Article

Recent Progress in Sodium Alginate-based Hydrogels Enriched With Magnetic Nanoparticles for Biomedical Applications: A Review

Kun Fang[1,2]*, Pei Li[3], Xiangrui Huang[1,2], Hanbing Wang [1,2], Yihan Li [1,2]

1 College of Tea and Food Science, Dabie Mountain Laboratory, Xinyang Normal University, Xinyang, Henan 464000, China
2 Henan Key Laboratory of Tea Plant Biology, Xinyang Key Laboratory of Food Innovation for Tea and Camellia oleifera , Xinyang, Henan 464000, China
3 Huaihe Campus Administrative Committee, Xinyang Normal University, Xinyang, Henan 464000, China
* Corresponding authors, E-mail address: fangkun@xynu.edu.cn (Kun Fang)

Abstract

The emergence of organic-inorganic hybrid composites integrating magnetic nanoparticles (MNPs) with polymers has been an important advancement in modern biological research. Among these systems, magnetic sodium alginate (SA)-based hydrogels (MSABHs), produced by embedding MNPs within an SA framework, exhibit remarkable potential for biomedical applications owing to their high biocompatibility, rapid magnetic response, controllable spatiotemporal behavior, and remote, non-invasive operation. Under the influence of an alternating magnetic field (AMF), MSABHs can exhibit various responses, including deformation, motion, and thermal generation, which are highly valuable for diagnostic and therapeutic medical applications. This review first outlines the key studies on SA and MNPs, along with the various synthesis routes used to fabricate MSABHs. Subsequently, the discussion primarily focuses on their versatile biomedical applications, including tissue engineering, targeted drug delivery, thermotherapy, imaging, and micro-robotics, followed by an evaluation of current challenges and prospects for future improvement. Through this comprehensive examination and synthesis, the review aims to further reveal the full potential of MSABHs and broaden their applications in the biological domain.

Keywords: Sodium alginate; Magnetic nanoparticles; Hydrogels; Biomedical applications

Academic Editor: Firstname Lastname

Received: date
Revised: date
Accepted: date
Published: date

Citation: To be added by editorial staff during production.

Copyright: © 2025 by the authors. Submitted for possible open access publication under the terms and conditions of the Creative Commons Attribution (CC BY) license (https://creativecommons.org/licenses/by/4.0/).

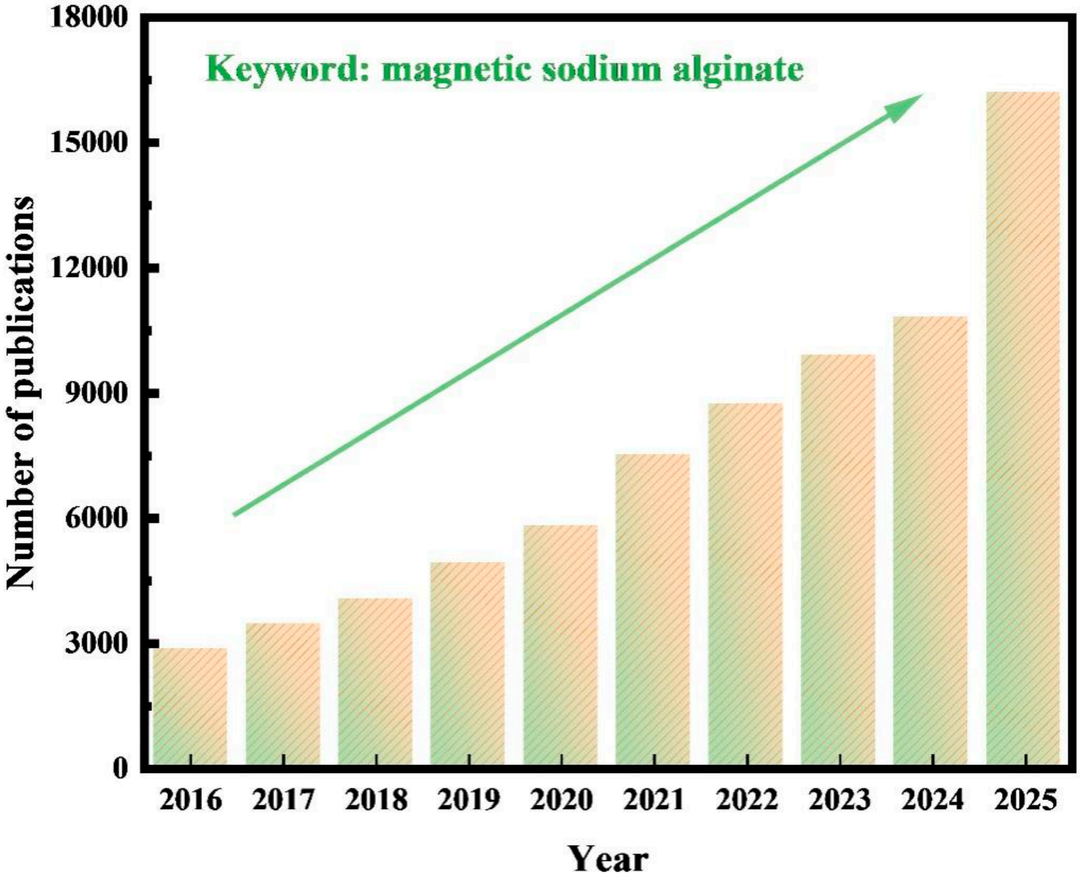

Figure S1. Scientific literature trend chart for the keyword "magnetic SA biomedical" retrieved via Google Scholar, with data available through Dec. 31, 2025.

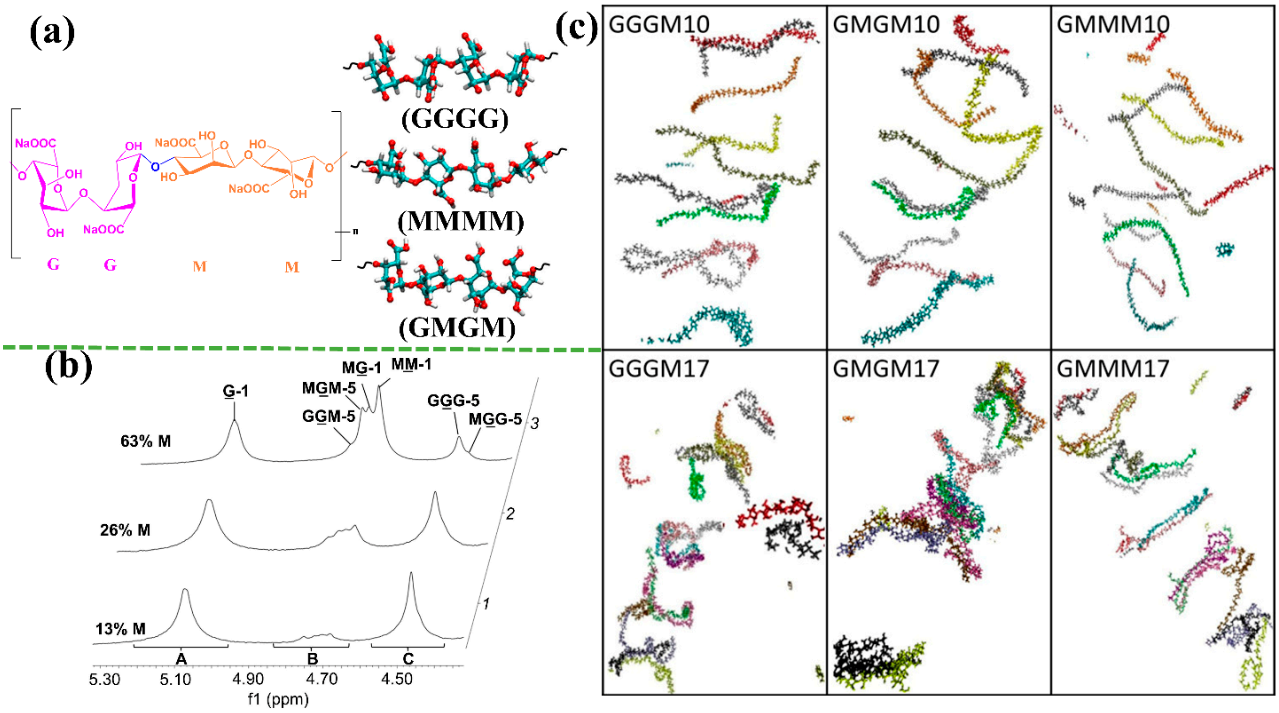

Figure S2. (a) Structural formulas and the lowest-energy conformers of GG, MM, and GM units generated with AVOGADRO and optimized using the GAFF force field. (b) Isomeric regions of the ^{1}H NMR spectra for alginate samples with varying M-block content, with peak assignments following the descriptions of Grasdalen [52]. Copyright 1983, Elsevier. (c) Snapshots of alginate chains (Nm = 30) in SA solution with alternating G/M monomer sequence GMGM, GMM, and GGM at different SA concentrations of Nc = 10 and 17. C. Reactive carboxy groups of SA [60]. Copyright 2016, ACS Publications.

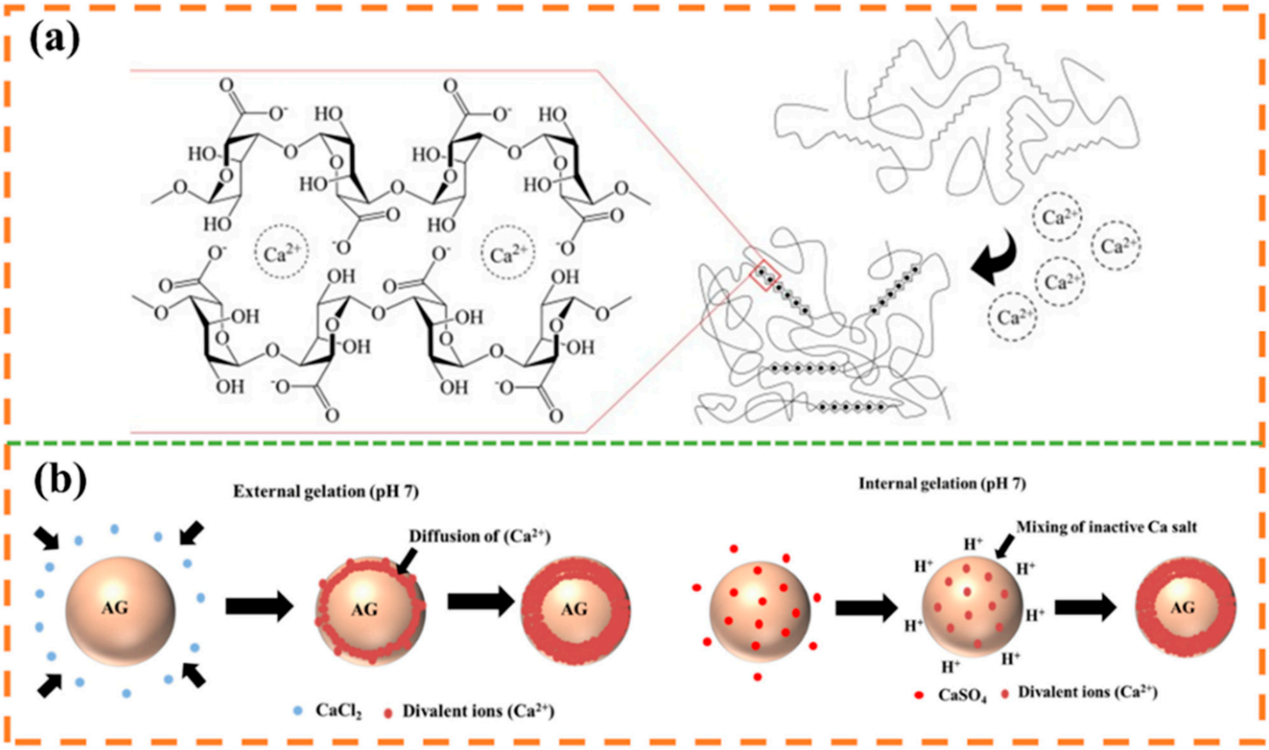

Figure S3. (a) SA gelation by calcium, the "egg-box" model [78]. Copyright 2020, Elsevier. (b) Alginate ionic gelation includes internal and external gelation [80]. Copyright 2022, Elsevier.

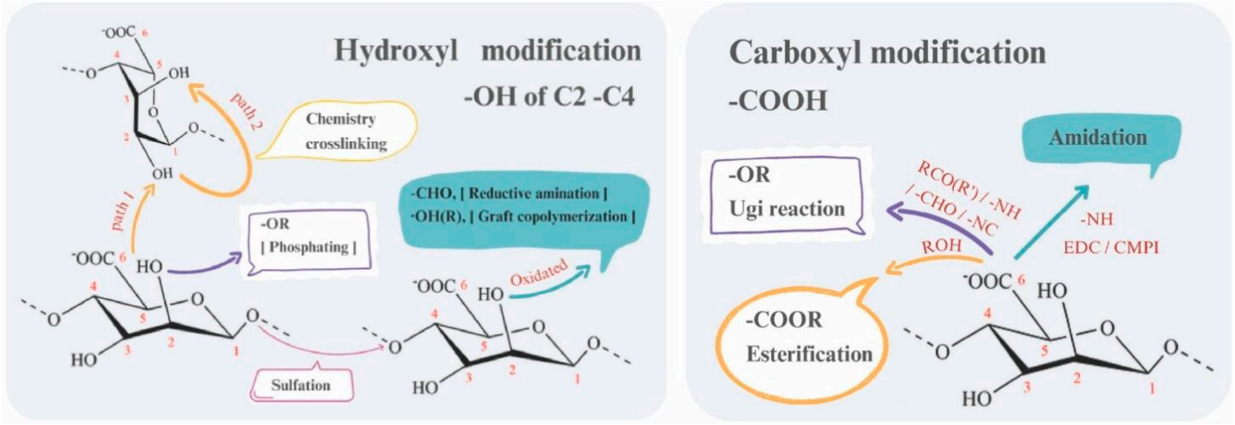

Figure S4. The point and mechanism of chemical modification [93]. Copyright 2024, Elsevier

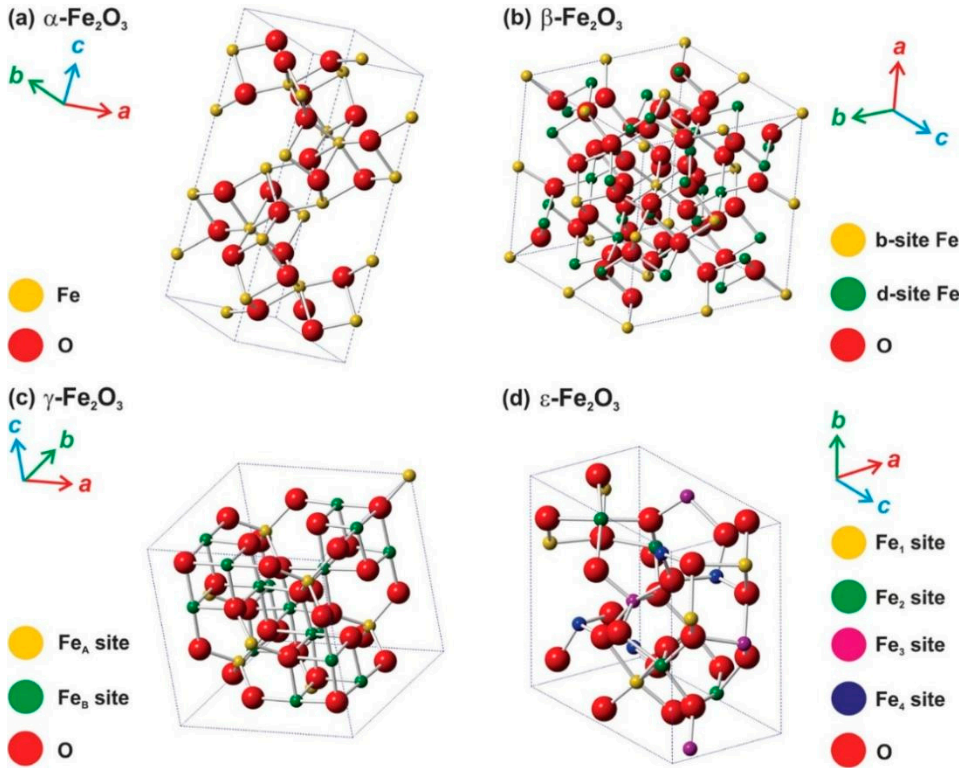

Figure S5. Fe_2O_3 fundamental crystal structures graphical representations: (a) α-Fe_2O_3, (b) β-Fe_2O_3, (c) γ-Fe_2O_3, (d) ε-Fe_2O_3 [101]. Copyright 2011, ACS publication.

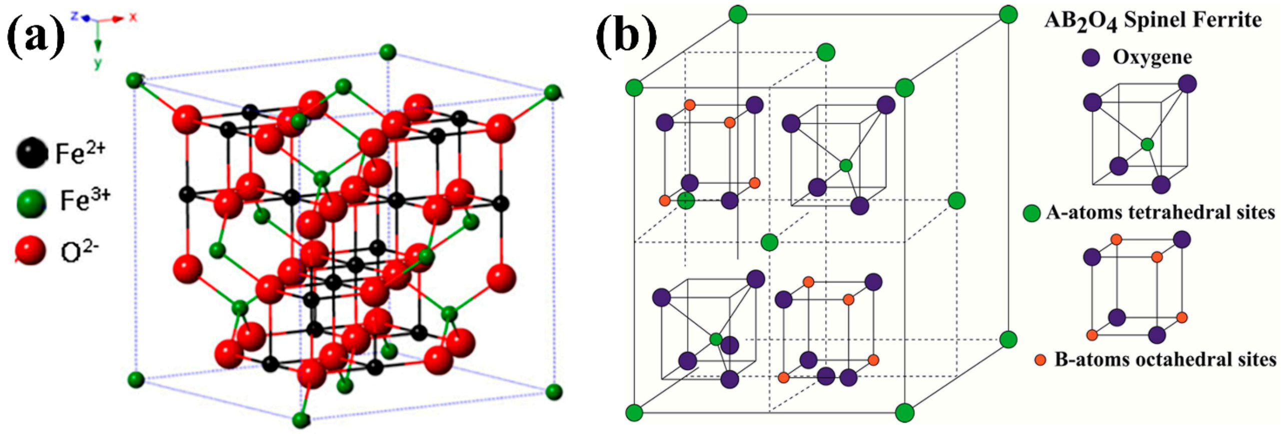

Figure S6. (a) Crystal structure of magnetite (Fe_3O_4) phase [102]. Copyright 2015, IOP Publishing; (b) Unit cell structure of magnetic spinel ferrite [103]. Copyright 2024, Elsevier.

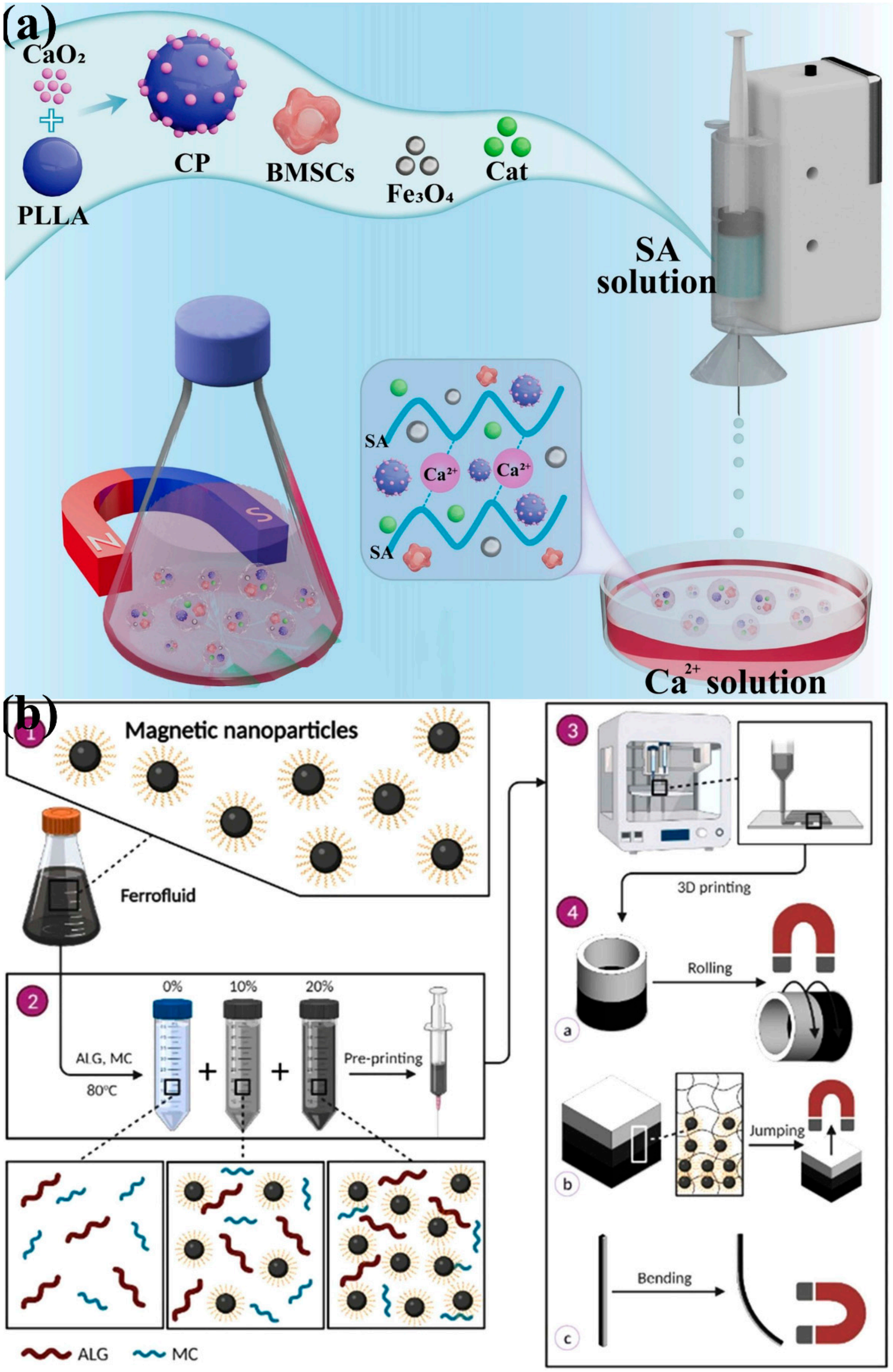

Figure S7. (a) Schematic representation of the preparation of magnetic SA microcarriers [106]. Copyright 2025, Elsevier. (b) Schematic representation of the fabrication of magnetically graded materials. (1) A ferrofluid composed of PAA-stabilized MNPs served as the starting material; (2) Magnetic inks were formulated with ferrofluid contents of 0%, 10%, and 20% (w/w); (3) These inks were then used for multimaterial 3D printing to construct magnetic structures; (4) The resulting patterned hydrogels exhibited distinct magnetic responses, enabling motions such as rolling (a), jumping (b), and bending (c) [107]. Copyright 2022, Elsevier.

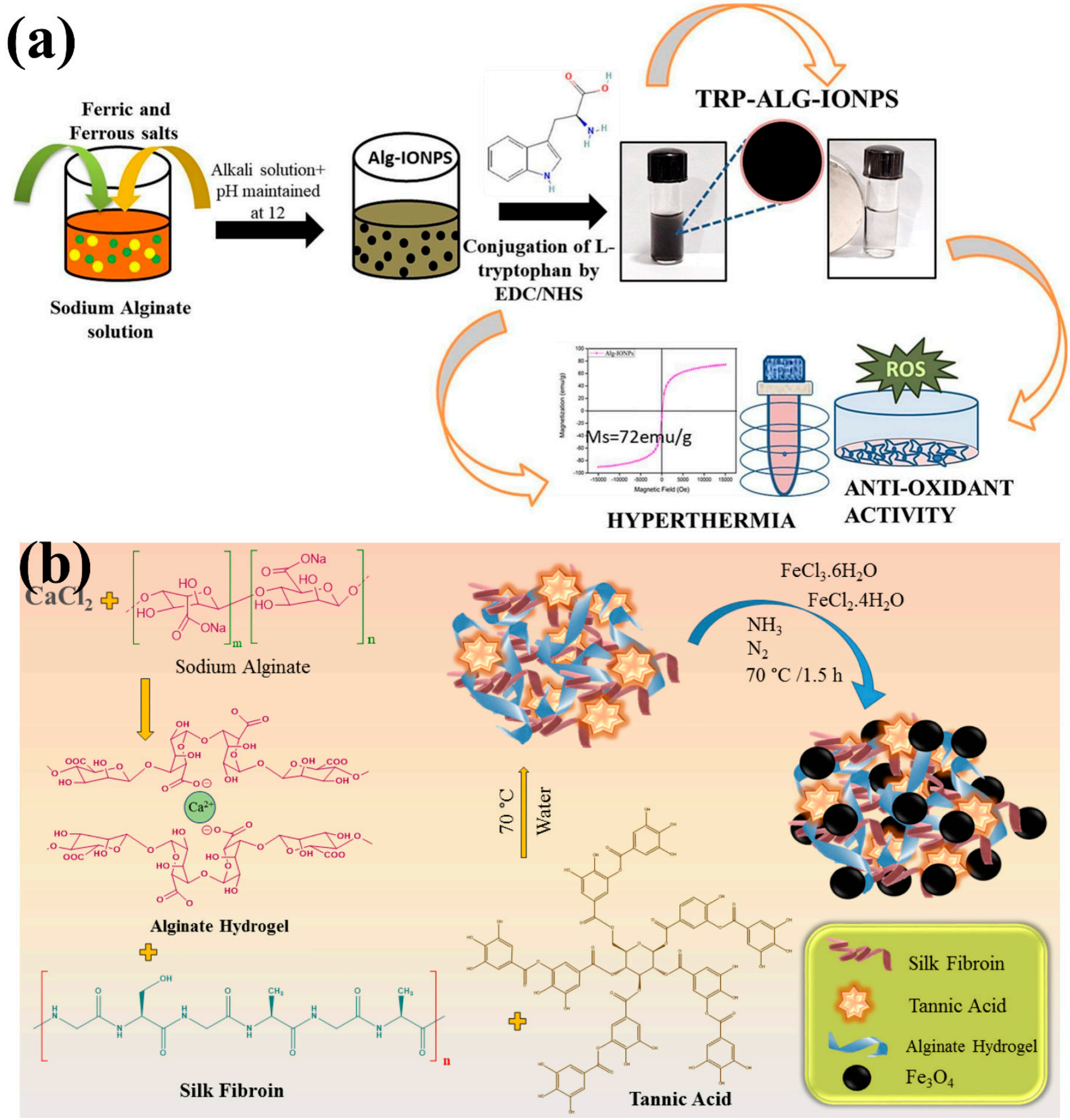

Figure S8. (a) Schematic representation of the fabrication process and evaluation of the synergistic potential of SA-Trp-Fe_3O_4 [109]. Copyright 2025, Elsevier. (b) Preparation of SA-TA hydrogel/SF/Fe_3O_4 magnetic nanocomposites [110]. Copyright 2023, Elsevier.

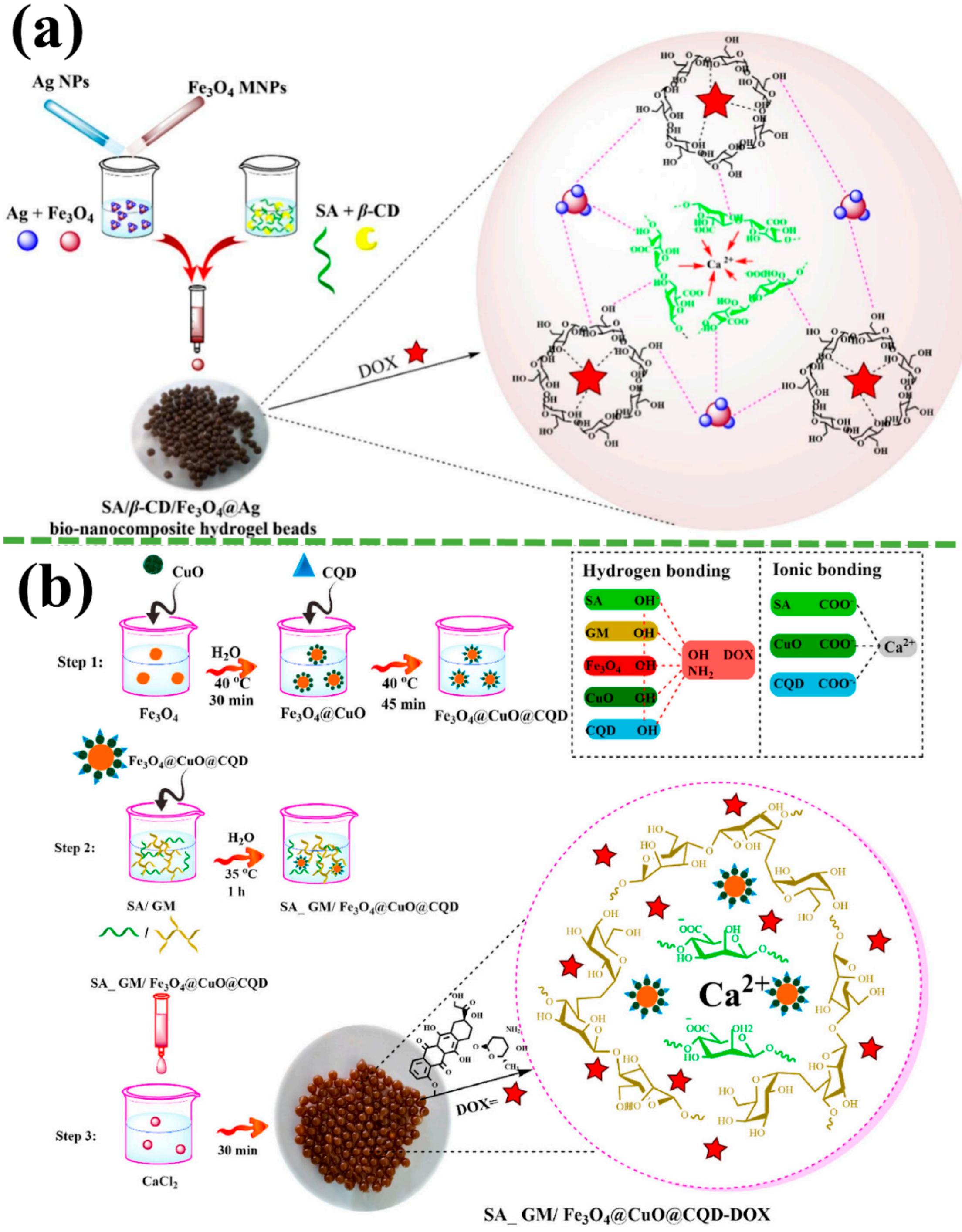

Figure S9. (a) Synthesis of SA/β-CD/Fe_3O_4@Ag MHBs [111]. Copyright 2025, Elsevier; (b) Synthesis of GM-SA/Fe_3O_4@CuO@CQD [112]. Copyright 2025, Elsevier.

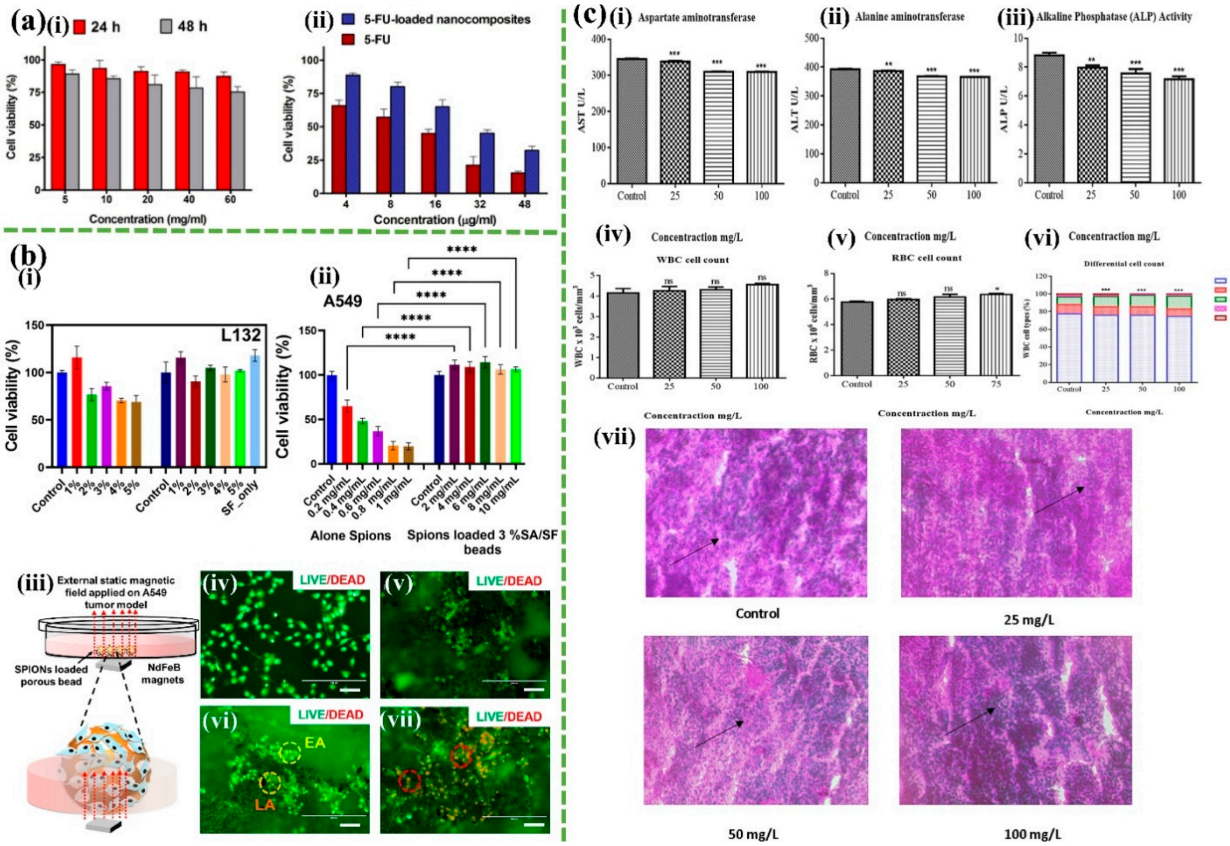

Figure S10. Toxicological evaluation of MSABHs. (a) (i) Cell viability of SA/HAp–Fe_3O_4after 24 and 48 h ($P < 0.05$); (ii) Viability of cells exposed to 5-FU alone and to 5-FU-loaded SA/HAp–Fe_3O_4at equivalent drug concentrations for 48 h ($P < 0.05$) [145]. Copyright 2025, Elsevier. (b) (i) Cytocompatibility of pure SA beads (1–5%) and silk-fibroin–incorporated SA beads (SA/SF, 1–5%); (ii) Viability analysis of bare MNPs (0.2–1.0 mg) and SF-containing SA beads (3% SA, lyophilized) loaded with MNPs at 2–10 mg; (iii) Illustration of the static magnetic field applied to cells cultured on MNP-embedded SA/SF porous beads; Live/dead staining of A549 cells: (iv) untreated control in standard culture plates; (v) cells within MNP-loaded porous beads without magnetic stimulation; (vi) cells exposed to a low static magnetic field (38 mT); (vii) cells subjected to a high static magnetic field (95 mT). Scale bar = 200 µm. Early apoptosis (EA) indicated by yellow dashed circles; late apoptosis (LA) by orange dashed circles; dead cells by red dashed circles [146]. Copyright 2023, Elsevier. (c) *In vivo* toxicology of FSE NCs. The results of liver enzyme AST(i), ALT (ii), and ALP (iii) in zebrafish blood were studied using iron oxide-SA nanocomposites supplemented with varying doses of eugenol (e.g., 25, 50, and 100 mg/L); (iv-vi) A study was conducted on adult zebrafish blood using iron oxide-SA nanocomposites supplemented with different doses of eugenol (e.g., 25, 50, and 100 mg/L); Analysis of the histopathological effects of eugenol in the liver tissue of adult zebrafish in FSE NCs [147]. Copyright 2024, Elsevier.
